# Patterns in health service use during and up to 1 year after an acute episode of hospital-presenting self-harm: data linkage cohort study of over 1.3 million records

**DOI:** 10.1192/bjo.2026.12033

**Published:** 2026-07-10

**Authors:** Katrina Witt, Daniel Z. Q. Gan, Caroline X. Gao, Jonathan Knott, Annette Erlangsen, Trine Madsen, Lianne Schmaal

**Affiliations:** Centre for Youth Mental Health, The University of Melbourne, Parkville, Australia; Orygen, Parkville, Australia; School of Public Health and Preventive Medicine, Monash University, Melbourne, Australia; Department of Critical Care, The University of Melbourne, Parkville, Australia; Emergency Department, Royal Melbourne Hospital, Parkville, Australia; Danish Research Institute for Suicide Prevention, Mental Health Center Copenhagen, Copenhagen, Denmark; Copenhagen Research Center for Mental Health, Mental Health Center Copenhagen, Copenhagen, Denmark; Centre for Mental Health Research, National Centre for Epidemiology and Population Health, The Australian National University, Canberra, Australia; Department of Mental Health, Johns Hopkins Bloomberg School of Public Health, Baltimore, Maryland, USA; Section for Epidemiology, Faculty of Health and Medical Sciences, University of Copenhagen, Copenhagen, Denmark

**Keywords:** Self-harm, suicide, mental health services, general adult psychiatry, register-based epidemiology

## Abstract

**Background:**

Many people presenting to emergency departments after self-harm do not receive adequate care, even in well-resourced health systems.

**Aims:**

To identify patterns of health care service use across two periods: (a) during and (b) up to 1 year after an index emergency department self-harm presentation.

**Method:**

A retrospective population-based cohort study including 4668 individuals aged ≥9 years who presented to the Royal Melbourne Hospital emergency department for self-harm between January 2012 and December 2019. Linked administrative data captured >1.3 million records across primary care, pharmacy, specialist mental and physical health services and emergency departments. Sequential pattern mining identified longitudinal service-use clusters. Multinomial regression explored associations with demographic, clinical, psychosocial and presentation characteristics. Cox proportional hazards models assessed associations between clusters and all-cause and suicide mortality.

**Results:**

Emergency department self-harm presentations triggered short-term increases in multi-sector contacts. However, most (68.7%) reverted to the same service-use cluster observed prior to their index presentation. Suicide risk was highest within 1 year, particularly among those in the specialist mental health services cluster (4.5% of the cohort).

**Conclusions:**

A small subgroup engage intensively with specialist mental health services yet remain at high suicide risk, while one in ten remain disengaged altogether, suggesting that an acute presentation of self-harm often fails to alter patients’ care trajectories long-term. Policy alignment with national recommendations for integrated, community-based care could improve sustained, evidence-based support beyond acute crises.

Persons presenting to emergency departments after non-fatal self-harm (i.e. any act of self-injury or self-poisoning irrespective of suicidal intent) are at high risk of repetition and premature mortality, including by suicide. The risk of self-harm repetition is highest in the first year following an index emergency department presentation for self-harm, with 16.3% re-presenting to emergency departments during this period.^1^ One in 25 of those who present to an emergency department after self-harm will die by suicide within 5 years, with most deaths occurring within the first year.^1^ Importantly, the risk of repeat self-harm in particular is not evenly distributed across this year. Prior studies have shown that up to one in ten who go on to repeat self-harm will do so within the first month post-discharge,^2–4^ a critical period during which the acute crisis that precipitated self-harm often remains unresolved. This observation supports focusing on the early post-discharge period, when interventions to prevent repeat self-harm and suicide may be most effective.

## Evidence-based treatment services within the mental health care sector

Evidence-based mental health treatments can reduce risks of self-harm repetition and, to a lesser extent suicide.^5^ However, many of those who present to emergency departments after self-harm do not receive adequate follow-up mental health care. Globally, around two-thirds of those who present to emergency departments after self-harm receive a mental health assessment in the department,^6^ whereas only one-in-six receive at least one out-patient mental health treatment contact.^7^ Repeat presentations are even less likely to result in either a psychosocial assessment, referral and/or receipt of specialist mental health care, and these proportions have not improved over time.^6,7^

## Treatment services beyond the mental health care sector

Beyond the mental health sector, the temporal dynamics of service use in the critical intervention window encompassing up to one year after self-harm remain poorly understood. It is unclear, for example, whether increased contact with primary care and hospital services reflects system responses to changing individual risk, or whether these different patterns influence subsequent self-harm or suicide risk. Using linked administrative data spanning primary care, pharmacy, hospital and specialist services, we aimed to identify distinct patterns of health care contact across two periods: (a) during the acute self-harm episode and (b) up to one year after the index emergency department self-harm presentation. We examine whether demographic, clinical or emergency department presentation characteristics predict engagement with mental health versus other services, and whether these patterns are differentially associated with risks of death, including by suicide. To our knowledge, no study has examined cross-sector health service use trajectories after self-harm using sequential pattern mining.

## Method

### Population and study design

We used a retrospective cohort of all persons aged ≥9 years presenting with self-harm to the Royal Melbourne Hospital (RMH) emergency department from 1 January 2012 to 31 December 2019. The RMH emergency department serves a primary catchment area of 1.6 million persons in metropolitan Melbourne, Victoria, Australia.^8^ Person identifiers were extracted by the RMH data custodian (J.K.) and provided to the Data Integration Services Centre (DISC). Using probabilistic matching, linkage was performed by the Australian Institute of Health and Welfare (AIHW) using the National Linkage Map, which covers 95% of the Australian population.

Cases were identified from the Self-Harm Monitoring System for Victoria.^9^ Data extracted included demographics, physical and psychiatric comorbidities, psychosocial factors and index emergency department presentation characteristics (e.g. alcohol involvement, arrival mode, mental health assessment, self-harm method). Self-harm methods were classified as per the Australian Bureau of Statistics (ABS) ICD-10 as: intentional drug overdose (X60–65), self-poisoning (X66–69), asphyxia/hanging (X70), drowning (X71), firearms/explosives (X72–75), self-cutting (X78) and other methods (X76, 77, 79–84 and Y87.0).

### Health care patterns

A care pattern is defined as sequences of care states within a fixed time period.^10^ We distinguished between two primary periods: (a) during an acute self-harm episode (defined as a 28-day window from the date of the index self-harm presentation. Where an individual had multiple presentations within this 28-day period, these were treated as one episode); and (b) up to one year after the acute self-harm episode. Seven care states were defined: (a) community services; (b) emergency departments; (c) general practitioners (GPs); (d) pharmacy; (e) specialist alcohol and other drug (AOD) services; (f) specialist mental health services and (g) specialist physical health services (Table 1, Supplementary Document). Analyses of service use in the year preceding the index self-harm presentation were also conducted and are presented in the Supplementary Document to allow comparison with post-crisis trajectories (see Supplementary Tables SD2, SD3 and Figs SD7–SD9).

**Table 1:** Classification of health service contacts by care state and data source Table 1 long description.

Care state	Data source(s) and definitions
Community services	Mental Health Community Support Services (MHCSS) Community Health Minimum Dataset (CHMDS)
Emergency departments	Victorian Emergency Minimum Dataset (VEMD)
General practitioners	Medicare Benefits Scheme (MBS)
Pharmacy	Pharmaceutical Benefits Scheme (PBS)
Specialist alcohol and other drug (AOD) services	Alcohol and Drug Information System (ADIS), prior to 2017 Victorian Alcohol and Drug Collection (VADC), post–2017
Specialist mental health services	Victorian Admitted Episodes Dataset (VAED) Victorian Integrated Non-Admitted Health (VINAH) Operational Data Store (ODS) for any contact coded as ‘mental health’ and/or with mental health providers (e.g. psychologists, psychiatrists)
Specialist physical health services	Victorian Admitted Episodes Dataset (VAED) Victorian Integrated Non-Admitted Health (VINAH) Operational Data Store (ODS) for any contact coded as ‘physical health’ and/or with physical health providers (e.g. dentists, physiotherapists)

These data-sets comprise administrative health service use records rather than discrete clinical encounters. Each record therefore reflects a health system transaction. Multiple records may therefore relate to a single clinical episode of care.

### Outcomes

Dates and cause of death were obtained from the Victorian Deaths Index (VDI), the National Deaths Index (NDI) and the Cause of Death Unit Record File (CODURF). Deaths by by suicide were defined using ICD-10 codes: X60 to X84 and Y87.0.

### Statistical analyses

To describe the typical duration and spacing of care between successive health care records, for each period, we calculated the median and interquartile range (IQR) of treatment days between successive treatment contacts. For prescriptions, we calculated the interval between prescription and supply dates to provide insight into potential delays in accessing pharmacological treatment.

For the purposes of analysis, each administrative record was mapped to one of the seven care states defined in Table 1. Records that could not be classified were coded as missing. Individual health care sequences were analysed using sequential pattern mining which is a data-driven method that can identify frequently occurring ordered sequences of events across individuals. Unlike cross-sectional summaries, which only provide static snapshots, this method captures dynamic changes in the temporal order in which health care contacts occur after an event. In the context of health care research, this method can help uncover typical pathways through the system. Sequences were visualised with sequence index and state distribution plots.

Clusters were derived via optimal matching and hierarchical agglomerative clustering. The optimal number of clusters to retain was determined through visual inspection of elbow plots, silhouette widths, within-cluster dissimilarity and minimum cluster size (≥100). To group individuals with similar patterns into these clusters we next quantified the dissimilarity between each individual’s pattern using optimal matching with substitution costs derived from observed transition rates. This means that clinically, each cluster represents a group of patients whose sequences of health care contact share similar structure and temporal order. Univariate multinomial logistic regression models examined associations between cluster membership and demographic, clinical, psychosocial or presentation factors for each period.

Finally, survival models assessed associations between cluster membership (exposure) and mortality. Cox proportional hazards models estimated hazard ratios and 95% CIs for all–cause mortality. Fine–Gray subdistribution hazards models estimated subdistribution hazard ratios and 95% CIs for suicide mortality, accounting for competing risks. Individuals were followed from the index presentation until death or end of follow-up (31 December 2020), whichever occurred first.

Analyses were undertaken in R version 4.5.2 for Windows (R Foundation for Statistical Computing, Vienna, Austria; see: https://www.R-project.org/), using the *TraMineR*, *nnet, survival* and *cmprsk* packages.

### Ethics

Ethical approval was granted by the Human Research Ethics Committees (HREC) of both Melbourne Health (2020.066) and the AIHW (EO2022-4-1354) with a retrospective waiver of consent.

## Results

Between 2012 and 2019, 551 692 emergency department presentations occurred, 7737 (1.4%) for non-fatal self-harm. The most common method was intentional drug overdose (*n* = 4678; 60.5%). Of these, the most commonly ingested medications were diazepam (*n* = 934; 20.0%), paracetamol (*n* = 765; 16.4%) and quetiapine (*n* = 467; 10.0%). One-quarter were for self–cutting (*n* = 2075; 26.8%) involving the forearm (*n* = 342; 16.5%). The bodily region involved, however, was not recorded for most self-cutting presentations (*n* = 1282; 61.8%). These presentations were by 5378 unique persons (54.7% female). Most (*n* = 4500; 83.7%) had only one presentation over the study period, giving an overall repetition rate of 16.3% (*n* = 878). Among those with multiple presentations, the median number was 2 (IQR 2 to 3).

**Fig. 1:**
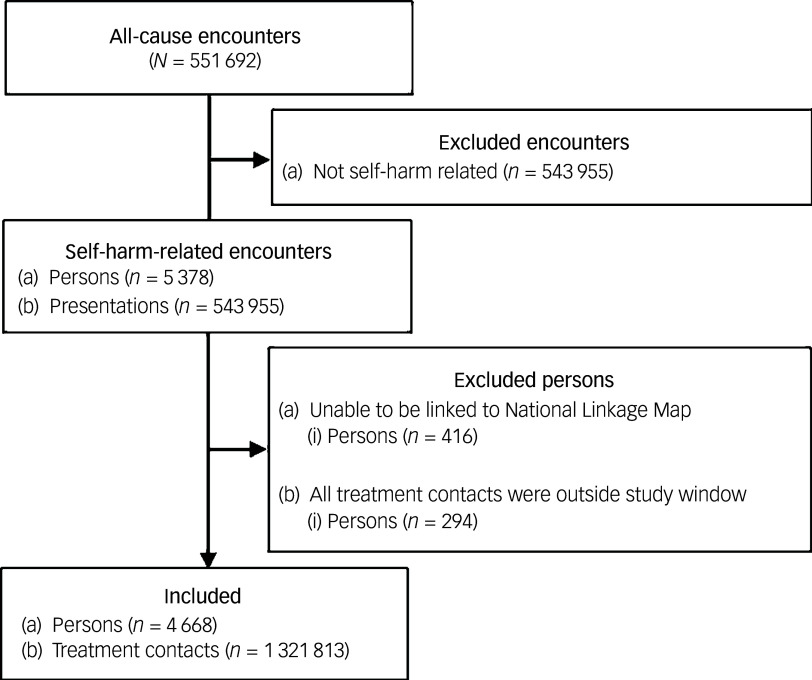
Fig. 1 long description. Selection of unique persons, and their accompanying treatment contacts, included in all subsequent analyses.

Records for 416 (7.7%) persons could not be linked to the National Linkage Map, primarily due to missing address information. The majority of these were female, younger, had a shorter emergency department stay, either self-presented or were transported by police to the emergency department, were less likely to be transported by road ambulance and more likely to be discharged home. Importantly, there were no significant differences by method of self-harm, alcohol co-involvement or presentation seriousness – as measured by the Australasian Triage Scale – or on likelihood of being assessed by emergency mental health staff in the emergency department (Supplementary Table SD1). Given the small proportion of unlinked records and the similarity on key clinical and service-use characteristics, missing data due to linkage is unlikely to bias outcome associations. A further 294 (5.5%) were excluded because treatment contacts occurred outside the study observation window. This left 4668 (86.7%) unique persons (representing 1 321 813 (98.5%) records) with available data for pattern mining and clustering (Fig. 1).

### Treatment service patterns during acute self-harm presentations

Overall, 1015 persons (21.7%) had no service use record other than their index self-harm presentation in this period, whereas 3653 persons (78.3%; 55.1% females) had at least 1 record (median 3, IQR 2 to 8). For these persons, the median duration of treatment was 1 day (IQR 1 to 2), principally as most records during this period were with the pharmacy (Table 2). For those with multiple service use records during this period, the median duration between contact was 2 days (IQR 1 to 3).

**Table 2:** Summary of treatment contacts by service and provider during and up to one year after an index episode of non–fatal self–harm presenting to the emergency department of the Royal Melbourne Hospital, 1 January 2012 to 31 December 2019 Table 2 long description.

Treatment service and provider	During		After	
	Contacts (%)	Persons (%)	Contacts (%)	Persons (%)
Treatment service				
Community services	33 (0.8)	27 (0.9)	304 (1.1)	99 (1.7)
Emergency departments	379 (8.7)	311 (10.7)	2969 (10.3)	1231 (20.8)
General practice	421 (9.7)	365 (12.6)	2803 (9.7)	1148 (19.4)
Pharmacy	1846 (42.4)	1093 (37.7)	12 898 (44.8)	1518 (25.7)
Specialist mental health services	648 (14.9)	362 (12.5)	2389 (11.5)	442 (7.5)
Specialist alcohol and other drug services	38 (0.9)	32 (1.1)	347 (1.2)	161 (2.7)
Specialist physical health services	968 (22.2)	692 (23.9)	5990 (20.8)	1264 (21.4)
Unknown/not reported	18 (0.4)	16 (0.6)	161 (0.6)	45 (0.8)
Treatment provider				
Addictions medicine specialist	58 (0.7)	26 (0.9)	590 (1.0)	140 (2.0)
Allied health worker	1020 (11.7)	426 (14.0)	5496 (9.5)	1008 (14.1)
Community/voluntary worker	<10	<10	20 (0.0)	<10
Counsellor	48 (0.5)	21 (0.7)	446 (0.8)	83 (1.2)
Dentist	–	–	12 (0.0)	<10
Doctor, not otherwise specified	496 (5.7)	205 (6.7)	3598 (6.2)	592 (8.3)
Emergency medicine specialist	758 (8.7)	311 (10.2)	5938 (10.2)	1231 (17.2)
General practitioner	842 (9.6)	365 (12.0)	5608 (9.7)	1148 (16.0)
Indigenous health worker	<10	<10	16 (0.0)	<10
Nurse	54 (0.6)	20 (0.7)	362 (0.6)	69 (1.0)
Occupational therapist	52 (0.6)	21 (0.7)	486 (0.8)	65 (0.9)
Pharmacist	3692 (42.2)	1093 (35.9)	25 796 (44.5)	1518 (21.2)
Physician	182 (2.1)	82 (2.7)	1410 (2.4)	381 (5.3)
Psychologist/psychiatrist	1302 (14.9)	365 (12.0)	6590 (11.4)	448 (6.3)
Social worker	28 (0.3)	12 (0.4)	150 (0.3)	31 (0.4)
Surgeon	138 (1.6)	64 (2.1)	882 (1.5)	263 (3.7)
Discipline not stated	60 (0.7)	27 (0.9)	578 (1.0)	167 (2.3)

Six distinct clusters were identified (Supplementary Fig. SD1–SD3): (a) pharmacy and specialist AOD services (729 persons, 34.0%); (b) specialist physical health services (496 persons); (c) specialist mental health services (318 persons, 14.8%); (d) GPs (247 persons); (e) pharmacy (200 persons, 9.3%) and (f) emergency departments (153 persons, 7.1%) (Supplementary Figs SD1 and SD2).

Compared with the pharmacy cluster (reference), cardiovascular disease (CVD), diabetes and alcohol involvement were associated with assignment to the physical health cluster. Substance use disorder, housing or legal problems, alcohol co-involvement and use of self-cutting or self-poisoning were associated with assignment to the mental health cluster, whereas female sex was associated with a reduced likelihood of assignment to this cluster. Younger age and use of self-cutting were associated with assignment to the primary care cluster, wheres arriving by road ambulance was associated with a reduced likelihood of assignment to this cluster. And younger age, experiencing housing or legal problems, and trauma exposure were all associated with assignment to the emergency department cluster, whereas female sex was associated with a lower likelihood of being assigned to this cluster (Table 3).

**Table 3:** Univariate multinomial regression models predicting service use pattern clusters during an episode of non-fatal self-harm presenting to the emergency department of the Royal Melbourne Hospital Table 3 long description.

Factor	1: Pharmacy + AOD services cluster				2: Specialist physical health services cluster				3: Specialist mental health services cluster				4: Primary care cluster				5: Pharmacy cluster (reference)				6: Emergency department cluster			
	*n*	OR	(95% CI)	*p*	*n*	OR	(95% CI)	*p*	*n*	OR	(95% CI)	*p*	*n*	OR	(95% CI)	*p*	*n*	OR	(95% CI)	*p*	*n*	OR	(95% CI)	*p*
Demographic																								
Age group – youth	210	1.17	(0.82–1.67)	0.397	**157**	**1.46**	**(1.00–2.12)**	**0.048**	52	1.26	(0.84–1.89)	0.259	**106**	**2.02**	**(1.35–3.04)**	**0.001**	52	1.00	(1.00–1.00)	NA	**58**	**1.78**	**(1.12–2.82)**	**0.014**
ATSI background	<10	–	–	–	<10	–	–	–	<10	–	–	–	<10	–	–	–	<10	1.00	(1.00–1.00)	NA	<10	–	–	–
Sex – female	418	0.81	(0.58–1.11)	0.190	140	0.65	(0.46–0.90)	0.106	**125**	**0.47**	**(0.33–0.68)**	**<0.001**	154	0.99	(0.68–1.46)	0.974	<10	1.00	(1.00–1.00)	NA	**65**	**0.44**	**(0.28–0.68)**	**<0.001**
Usual place of residence – regional/rural	55	0.89	(0.51–1.58)	0.697	31	0.72	(0.39–1.33)	0.287	31	1.18	(0.63–2.19)	0.606	17	0.78	(0.39–1.57)	0.487	17	1.00	(1.00–1.00)	NA	<10	–	–	–
Physical																								
No recorded physical illness (reference)	–	–	–	–	–	–	–	–	–	–	–	–	–	–	–	–	–	–	–	–	–	–	–	–
Cancer	27	0.82	(0.38–1.77)	0·606	39	1.81	(0.86–3.81)	0.118	12	0.83	(0.34–2.01)	0.684	<10	–	–	–	<10	1.00	(1.00–1.00)	NA	<10	–	–	–
Chronic pain	43	1.50	(0.69–3.25)	0.230	35	1.82	(0.83–4.00)	0.135	13	1.02	(0.42–2.51)	0.961	<10	–	–	–	<10	1.00	(1.00–1.00)	NA	<10	–	–	–
CVD	50	1.56	(0.75–3.23)	0.229	**59**	**2.86**	**(1.39–5.90)**	**0.004**	24	1.73	(0.78–3.81)	0.171	10	0.90	(0.36–2.25)	0.814	<10	1.00	(1.00–1.00)	NA	10	1.48	(0.59–3.75)	0.404
Diabetes	40	1.60	(0.71–3.63)	0.260	**50**	**3.09**	**(1.38–6.94)**	**0.006**	25	2.35	(0.98–5.55)	0.051	10	1.16	(0.43–3.11)	0.763	<10	1.00	(1.00–1.00)	NA	<10	–	–	–
Psychiatric																								
Anxiety disorder (any)	16	1.10	(0.36–3.33)	0.866	<10	–	–	–	<10	–	–	–	<10	–	–	–	<10	1.00	(1.00–1.00)	NA	<10	–	–	–
Mood disorder (any)	78	1.14	(0.67–1.93)	0.623	31	0.64	(0.35–1.15)	0.136	40	1.37	(0.77–2.44)	0.284	25	1.07	(0.57–2.01)	0.826	19	1.00	(1.00–1.00)	NA	18	1.27	(0.64–2.51)	0.492
Eating disorder (any)	–	–	–	–	–	–	–	–	–	–	–	–	–	–	–	–	–	–	–	–	–	–	–	–
Psychotic disorder (any)	<10	–	–	–	<10	–	–	–	<10	–	–	–	<10	–	–	–	<10	1.00	(1.00–1.00)	NA	<10	–	–	–
Personality disorder (any)	10	0.68	(0.21–2.20)	0.521	<10	–	–	–	10	1.59	(0.49–5.14)	0.438	<10	–	–	–	<10	1.00	(1.00–1.00)	NA	<10	–	–	–
Substance use disorder (any)	284	1.16	(0.84–1.61)	0.373	172	0.96	(0.68–1.36)	0.837	**141**	**1.45**	**(1.01–2.08)**	**0.047**	72	0.75	(0.50–1.11)	0.152	71	1.00	(1.00–1.00)	NA	60	1.17	(0.76 – 1.81)	0.474
Psychosocial																								
Exposure to family violence	11	3.05	(0.39–23.77)	0.287	10	4.10	(0.52–32.22)	0.180	<10	–	–	–	<10	–	–	–	<10	1.00	(1.00–1.00)	NA	<10	–	–	–
Exposure to traumatic experiences	64	1.19	(0.66–2.13)	0.566	54	1.51	(0.83–2.74)	0.178	39	1.72	(0.92–3.22)	0.087	15	0.80	(0.38–1.67)	0.549	15	1.00	(1.00–1.00)	NA	**29**	**2.88**	**(1.49–5.60)**	**0.002**
Housing problems	12	3.33	(0.43–25.79)	0.239	<10	–	–	–	**14**	**9.17**	**(1.20–70.31)**	**0.033**	<10	–	–	–	<10	1.00	(1.00–1.00)	NA	**<10**	–	–	–
Legal problems	68	1.18	(0.67–2.09)	0.562	47	1.20	(0.67–2.18)	0.540	**46**	**1.94**	**(1.07–3.54)**	**0.029**	15	0.74	(0.36–1.54)	0.426	16	1.00	(1.00–1.00)	NA	**30**	**2.80**	**(1.47–5.36)**	**0.002**
Refugee background	–	–	–	–	–	–	–	–	–	–	–	–	–	–	–	–	–	–	–	–	–	–	–	–
Relationship problems – partner	–	–	–	–	–	–	–	–	–	–	–	–	–	–	–	–	–	–	–	–	–	–	–	–
Relationship problems – any other person	–	–	–	–	–	–	–	–	–	–	–	–	–	–	–	–	–	–	–	–	–	–	–	–
Presentation characteristics (index episode)																								
Age at index episode (years)	NA	0.96	(0.98–1.01)	0.442	NA	1.00	(0.99–1.01)	0.897	NA	0.96	(0.98–1.00)	0.349	NA	**0.97**	**(0.95–0.98)**	**<0.001**	NA	1.00	(1.00–1.00)	NA	NA	**0.98**	**(0.96–0.99)**	**0.005**
Alcohol co-involvement	**180**	**0.67**	**(0.47–0.93)**	**0.019**	**106**	**0.55**	**(0.38–0.70)**	**0.001**	**67**	**0.54**	**(0.36–0.81)**	**0.003**	66	0.74	(0.49–1.11)	0.148	66	1.00	(1.00–1.00)	NA	41	0.74	(0.47–1.18)	0.210
Arrival mode – self-presentation (reference)	98	–	–	–	86	–	–	–	42	–	–	–	60	–	–	–	27	–	–	–	25	–	–	–
Arrival mode – air ambulance	–	–	–	–	–	–	–	–	–	–	–	–	–	–	–	–	<10	1.00	(1.00–1.00)	NA	<10	–	–	–
Arrival mode – road ambulance	512	0.99	(0.62–1.58)	0.978	330	0.73	(0.45–1.17)	0.193	219	0.99	(0.58–1.68)	0.974	**152**	**0.48**	**(0.29–0.80)**	**0.005**	142	1.00	(1.00–1.00)	NA	99	0.75	(0.41–1.37)	0.355
Arrival mode – police facilitated	18	1.24	(0.39–3.97)	0.718	11	0.86	(0.25–2.93)	0.814	15	2.41	(0.72–8.04)	0.152	<10	–	–	–	<10	1.00	(1.00–1.00)	NA	<10	–	–	–
Arrival mode – not reported	95	1.01	(0.55–1.85)	0.983	61	0.74	(0.39–1.38)	0.342	33	0.82	(0.40–1.65)	0.572	31	0.54	(0.27–1.07)	0.078	26	1.00	(1.00–1.00)	NA	22	0.91	(0.42–2.01)	0.822
Self-harm method – IDO (reference)	317	–	–	–	177	–	–	–	177	–	–	–	155	–	–	–	143	–	–	–	91	–	–	–
Self-harm method – alcohol overdose	–	–	–	–	–	–	–	–	–	–	–	–	–	–	–	–	–	–	–	–	–	–	–	–
Self-harm method – drowning	–	–	–	–	–	–	–	–	–	–	–	–	–	–	–	–	–	–	–	–	–	–	–	–
Self-harm method – firearms/explosives	–	–	–	–	–	–	–	–	–	–	–	–	–	–	–	–	–	–	–	–	–	–	–	–
Self-harm method – asphyxia/hanging	15	0.87	(0.31–2.44)	0.797	15	1.35	(0.48–3.79)	0.565	12	1.94	(0.67–5.63)	0.224	<10	–	–	–	<10	1.00	(1.00–1.00)	NA	<10	–	–	–
Self-harm method – self-cutting	150	1.09	(0.74–1.62)	0.662	97	1.09	(0.72–1.66)	0.674	**79**	**1.60**	**(1.03–2.48)**	**0.037**	**69**	**1.59**	**(1.01–2.50)**	**0.043**	40	1.00	(1.00–1.00)	NA	41	1.61	(0.97–2.68)	0.066
Self-harm method – self-poisoning	12	3.49	(0.45–27.10)	0.231	13	5.86	(0.76–45.24)	0.090	**<10**	–	–	–	<10	–	–	–	**<10**	1.00	(1.00–1.00)	NA	<10	–	–	–
Self-harm method – other	33	1.92	(0.74–5.01)	0.182	**40**	**3.61**	**(1.40–9.34)**	**0.008**	**25**	**4.04**	**(1.51–10.82)**	**0.005**	<10	–	–	–	<10	1.00	(1.00–1.00)	NA	<10	–	–	–
Self-harm method – not reported	20	1.16	(0.43–3.16)	0.764	14	1.26	(0.44–3.57)	0.660	14	2.26	(0.80–6.43)	0.126	<10	–	–	–	<10	1.00	(1.00–1.00)	NA	<10	–	–	–
Seen by emergency mental health team in emergency department	383	1.02	(0.75–1.40)	0.893	235	0.83	(0.60–1.15)	0.270	180	1.20	(0.84–1.72)	0.305	137	1.15	(0.79–1.67)	0.465	104	1.00	(1.00–1.00)	NA	65	0.68	(0.45–1.04)	0.077

AOD, alcohol and other drug; ATSI, Aboriginal and/or Torres Strait Islander; CVD, cardiovascular disease; IDO, intentional drug overdose; OR, odds ratio; NA, not applicable. Dashes indicate factors with no data and/or reference categories. Odds ratios for groups less than ten suppressed as per Australian Institute of Health and Welfare privacy guidelines. Boldface indicates *p* is significant at conventional alpha 0.05 level.

### Treatment service patterns up to 1 year after self-harm

A total of 593 (12.7%) persons had no service use records in the year following their index presentation for self-harm, whereas 4075 (87.3%) had at least 1 during this period (median 25, IQR 9 to 64), mostly with pharmacy, followed by emergency departments and GPs (Table 2). Half of those with at least one service use record in this period (54.4%) were female. The median duration was 8 days (IQR 3 to 15), with a median of 10 days between consecutive records (IQR 4 to 20).

**Fig. 2:**
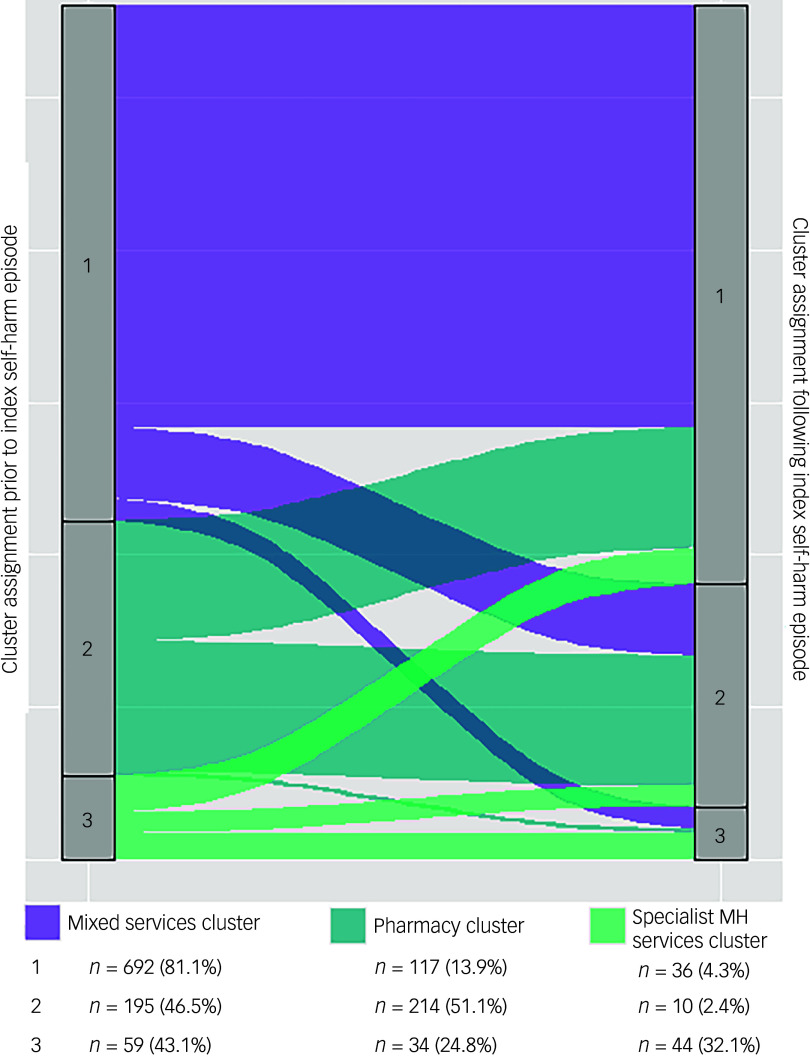
Fig. 2 long description. Sankey diagram depicting continuity of treatment contact cluster assignment for persons with at least one contact in the year before and after the index emergency department presentation for self–harm (*n* = 1401). MH, mental health.

Three distinct clusters were identified (Supplementary Fig. SD4). Just over one-third (1493 persons) were assigned to the specialist physical health services cluster, followed by the pharmacy cluster (626 persons, 15.4%) and the mental health services cluster (185 persons, 4.5%) (Supplementary Fig. SD5 and SD6). Clustering in this period largely mirrored patterns for the period prior to self-harm (Supplementary Tables SD2 and SD3, Figs SD7 to SD9). Indeed, for those with service use records both before and after their index self-harm presentation (*n* = 1401), most (68.7%) reverted to the same cluster as observed prior to their index presentation for self–harm. This was particularly so for those assigned to the mental health services cluster (Fig. 2).

Compared with the pharmacy-only cluster (reference), self-poisoning, self-cutting or other self-harm methods were associated with assignment to the mixed services use cluster, as was younger age and legal problems. Conversely, being assessed by mental health staff in the emergency department and arriving by road ambulance were associated with a reduced likelihood of assignment to this cluster. Assignment to the specialist mental health services cluster was associated with use of asphyxia/hanging, self-cutting, self-poisoning or any other method of self-harm, as well as legal problems, substance use disorder and being seen by mental health staff in the emergency department. Only female sex was associated with a reduced likelihood of assignment to the specialist mental health services cluster (Table 4).

**Table 4:** Univariate multinomial regression models predicting service pattern clusters up to one year after an episode of non-fatal self-harm presenting to the emergency department of the Royal Melbourne Hospital

Factor	1: Mixed services cluster				2: Pharmacy cluster (Reference)				3: Specialist mental health services cluster			
	*n*	OR	(95% CI)	*p*	*n*	OR	(95% CI)	*p*	*n*	OR	(95% CI)	*p*
Demographic												
Age group – youth	**615**	**1.62**	**(1.32–1.97)**	**<0.001**	192	1.00	(1.00–1.00)	NA	**76**	**1.53**	**(1.09–2.15)**	**0.010**
ATSI background	16	1.69	(0.56–5.06)	0.353	<10	1.00	(1.00–1.00)	NA	<10	–	–	–
Sex – female	796	0.82	(0.69–1.01)	0.057	362	1.00	(1.00–1.00)	NA	**86**	**0.63**	**(0.46–0.88)**	**0.007**
Usual place of residence – regional/rural	103	1.07	(0.73–1.56)	0.728	41	1.00	(1.00–1.00)	NA	15	1.32	(0.71–2.44)	0.384
Physical												
No recorded physical illness (reference)	–	–	–	–	–	–	–	–	–	–	–	–
Cancer	45	1.05	(0.60–1.83)	0.864	18	1.00	(1.00–1.00)	NA	<10	–	–	–
Chronic pain	36	0.75	(0.43–1.30)	0.306	20	1.00	(1.00–1.00)	NA	<10	–	–	–
CVD	85	1.58	(0.99–2.53)	0.056	23	1.00	(1.00–1.00)	NA	<10	–	–	–
Diabetes	80	1.26	(0.80–1.96)	0.317	27	1.00	(1.00–1.00)	NA	<10	–	–	–
Psychiatric												
Anxiety disorder (any)	30	0.90	(0.47–1.70)	0.738	14	1.00	(1.00–1.00)	NA	<10	–	–	–
Mood disorder (any)	146	0.76	(0.57–1.02)	0.068	78	1.00	(1.00–1.00)	NA	23	1.00	(0.61–1.64)	0.992
Eating disorder (any)	<10	–	–	–	<10	1.00	(1.00–1.00)	NA	<10	–	–	–
Psychotic disorder (any)	<10	–	–	–	<10	1.00	(1.00–1.00)	NA	<10	–	–	–
Personality disorder (any)	22	0.61	(0.31– 1.18)	0.143	15	1.00	(1.00–1.00)	NA	<10	–	–	–
Substance use disorder (any)	485	1.03	(0.85–1.16)	0.755	199	1.00	(1.00–1.00)	NA	**80**	**1.63**	**(1.17–2.29)**	**0.004**
Psychosocial												
Exposure to family violence	18	0.75	(0.35–1.64)	0.472	10	1.00	(1.00–1.00)	NA	<10	1.71	(0.58–5.07)	0.332
Exposure to traumatic experiences	118	0.86	(0.62–1.19)	0.360	57	1.00	(1.00–1.00)	NA	22	1.35	(0.80–2.27)	0.263
Housing problems	27	1.13	(0.55–2.36)	0.735	10	1.00	(1.00–1.00)	NA	<10	1.71	(0.57–5.07)	0.332
Legal problems	**165**	**1.53**	**(1.09–2.15)**	**0.014**	47	1.00	(1.00–1.00)	NA	**23**	**1.75**	**(1.03–2.30)**	**0.038**
Refugee background	–	–	–	–	–	–	–	–	–	–	–	–
Relationship problems – partner	–	–	–	–	–	–	–	–	–	–	–	–
Relationship problems – any other person	–	–	–	–	–	–	–	–	–	–	–	–
Presentation characteristics (index episode)												
Age at index episode (years)	**NA**	**0.98**	**(0.97–0.99)**	**<0.001**	NA	1.00	(1.00–1.00)	NA	NA	0.99	(0.97–1.00)	0.050
Alcohol co-involvement	356	0.89	(0.71–1.10)	0.265	164	1.00	(1.00–1.00)	NA	**35**	**0.66**	**(0.44–0.99)**	**0.044**
Arrival mode – self-presentation (reference)	329	1.00	(1.00–1.00)	NA	89	1.00	(1.00–1.00)	NA	24	1.00	(1.00–1.00)	NA
Arrival mode – air ambulance	12	1.08	(0.30–3.52)	0.904	<10	1.00	(1.00–1.00)	NA	<10	–	–	–
Arrival mode – road ambulance	**903**	**0.57**	**(0.32**–**0.73)**	**<0.001**	432	1.00	(1.00–1.00)	NA	126	1.08	(0.44–1.77)	0.755
Arrival mode – police facilitated	39	0.59	(0.44–1.07)	0.084	18	1.00	(1.00–1.00)	NA	<10	–	–	–
Arrival mode – not reported	**207**	**0.67**	**(0.47–0.94)**	**0.021**	84	1.00	(1.00–1.00)	NA	25	1.10	(0.59–2.02)	0.761
Self-harm method – intentional drug overdose (reference)	897	1.00	(1.00–1.00)	NA	437	1.00	(1.00–1.00)	NA	95	1.00	(1.00–1.00)	NA
Self-harm method – alcohol overdose	–	–	–	–	–	–	–	–	–	–	–	–
Self-harm method – drowning	–	–	–	–	–	–	–	–	–	–	–	–
Self-harm method – firearms/explosives	–	–	–	–	–	–	–	–	–	–	–	–
Self-harm method – asphyxia/hanging	27	1.46	(0.68–3.13)	0.330	<10	1.00	(1.00–1.00)	NA	**12**	**6.13**	**(2.51–14.97)**	**<0.001**
Self-harm method – self-cutting	**375**	**1.41**	**(1.12–1.77)**	**0.004**	130	1.00	(1.00–1.00)	NA	**45**	**1.59**	**(1.06–2.39)**	**0.024**
Self-harm method – self-poisoning	**38**	**2.64**	**(1.17–5.97)**	**0.019**	<10	1.00	(1.00–1.00)	NA	**<10**	–	–	–
Self-harm method – other	**109**	**2.31**	**(1.45–3.67)**	**<0.001**	23	1.00	(1.00–1.00)	NA	**18**	**3.60**	**(1.87–6.93)**	**<0.001**
Self-harm method – not reported	41	1.25	(0.69–2.25)	0.460	16	1.00	(1.00–1.00)	NA	**<10**	–	–	–
Seen by emergency mental health team in emergency department	**734**	**0.70**	**(0.58–0.85)**	**<0.001**	363	1.00	(1.00–1.00)	NA	**124**	**1.47**	**(1.04–2.08)**	**0.028**

ATSI, Aboriginal and/or Torres Strait Islander; OR, odds ratio; NA, not applicable.

### Survival analysis

Overall, 107 persons (29·0% female) died over the 7.9-year study period, most (*n* = 41, 38.3%) from unknown causes. One-quarter of these deaths were suicides *(n* = 28, 26.2%). The median time between the last service use record and death was 188.5 days (IQR 8.3 to 599.8). For one-quarter, their last service provider before death was emergency medicine specialists (*n* = 27, 25.2%), followed by pharmacists (*n* = 23, 21.5%), allied health staff (*n* = 15, 14.0%) and psychologists/psychiatrists (*n* = 11, 10.3%).

Half (*n* = 50, 46.7%, 26.0% female) died within 1 year of their index self-harm presentation, one-third by suicide (*n* = 17, 34.0%) or unknown causes (*n* = 17, 34.0%). For these, the median time from last service use record to death was 9 days (IQR 1.3 to 82.8). For just under half (42.0%), their last provider before their death were emergency medicine specialists.

### All-cause mortality

Assignment to the mixed or specialist mental health services clusters had no significant effect on all-cause mortality, either by conclusion of the study period (mixed cluster: hazard ratio 1.06, 95% CI 0.68–1.66; mental health cluster: hazard ratio 1.74, 95% CI 0.92–3.31) or the within the first year post discharge (mixed cluster: hazard ratio 1.69, 95% CI 0.82–3.51; mental health cluster: hazard ratio 1.90, 95% CI 0.63–5.64). While there was some statistical evidence of departure from the proportional hazards assumption for the overall study period (*χ* ^2^ = 11.40, *df* = 2, *p* = 0.003), visual inspection of the Schoenfeld residuals indicated this was not substantial (see Supplementary Fig. SD10). The proportional hazards assumption was met for the 1-year follow-up period (*χ* ^2^ = 4.07, *df* = 2, *p* = 0.130)

### Suicide mortality

Assignment to the specialist mental health services cluster was associated with a higher sub-hazard of suicide (sHR 10.19, 95% CI 1.06–97.80). There was no effect for assignment to the mixed services cluster (sHR 5.47, 95% CI 0.72–41.80). When follow-up was extended over the 7.9-year study period, these associations were no longer statistically significant (mixed cluster: sHR 2.23, 95% CI 0.77–6.44; mental health cluster: sHR 3.43, 95% CI 0.86–13.70), suggesting that the excess risk was concentrated in the first year after the index self-harm presentation. Cumulative incidence plots showed consistent subdistribution hazards over time (see Supplementary Fig. SD11).

## Discussion

In this cohort of 4668 individuals comprising 1 321 813 service use records, distinct clusters of service use were observed during and up to 1 year following an index emergency department self-harm presentation. Yet, despite this representing a high-risk cohort, one in ten had no service use record in the year following their index self-harm presentation. This rose to one in five during the acute self-harm period itself. While these proportions are lower than previously reported,^11^ there nonetheless remains a substantial proportion who remain largely disconnected from health services during periods of elevated risk.

During the acute self-harm period, service use became differentiated with six distinct clusters emerging: around one-third had AOD services contact, one-quarter had physical health services contact and around one in ten had contact primarily with either mental health services, GPs, pharmacies or emergency departments. However, this differentiation largely diminished in the year following the index presentation, with most reverting to their pre-crisis cluster. This was particularly evident among those already connected to mental health services.

Physical health comorbidities were consistently associated with an increased likelihood of assignment to specialist physical health clusters. It is likely that at least some of these contacts may be for injuries that may represent sequelae of previous self-harm episodes.^12^ Females were less likely to be referred to specialist mental health care providers. Male sex has previously been associated with a greater likelihood of receiving psychiatric treatment, particularly on an in-patient basis.^13^ Given that males tend to use methods associated with greater lethality,^14^ our findings suggest that system-wide service-level factors (e.g. risk-based prioritisation) may influence treatment decisions after self-harm. Consistent with this explanation, we also found that use of high-lethality self-harm methods at the index presentation and assessment by mental health staff in the emergency department were both associated with an increased likelihood of assignment to specialist mental health clusters in the year following the index presentation.

Although 1-year and overall suicide rates in our cohort (0.4 and 0.6%, respectively) were lower than global estimates, risk was concentrated in the first year after the index self-harm presentation, consistent with previous work.^1^ This was particularly apparent within the specialist mental health services cluster, which accounted for up to one-quarter of first-year suicides despite representing only 4.5% of the cohort. This concentration of risk may partly reflect confounding by indication as those managed in specialist mental health services are likely to have more severe or complex presentations. However, when follow-up was extended to the full 7.9-year study period, these associations were no longer significant, indicating that the excess risk of suicide is concentrated shortly after discharge from the emergency department.^15^

### Clinical and policy implications

Acute health system responses to self-harm crises appear short-lived, suggesting that an acute presentation of self-harm often fails to alter patients’ care trajectories long-term. For example, one-third of individuals accessed AOD services during the acute self-harm episode, but brief contacts suggests limited intervention consisting of little more than a screening or assessment contact. Alcohol use disorder is a well-established risk factor for repeat self-harm^16^ and suicide,^17^ and longer-term (up to 6 months) interventions targeting harmful alcohol use can reduce self-harm and, to a lesser extent, suicide.^18^ Yet, mental health sector funding is seven times greater than for the AOD sector, even after accounting for differences in the burden of disease.^19^ In 2025, the Productivity Commission’s review of the National Mental Health and Suicide Prevention Agreement found that AOD services are inadequately embedded into mental health services, despite those experiencing AOD harms being explicitly identified as a priority population.^20^

Despite record levels of investment into specialist mental health care in Australia over recent decades (from AU$49.1 million in 2015–16^21^ to AU$14.5 billion in 2019–20),^22^ we also found that only a small proportion of those presenting to the emergency department after self-harm had contact with mental health services. Of these, one-third had previous contact with specialist mental health services and only 6.7% were newly represented in this cluster as a result of their self-harm presentation. This is concerning given that structured psychological interventions delivered in these settings, such as cognitive behavioural therapy (CBT) and dialectical behaviour therapy (DBT), remain the most evidence-based approaches for reducing repeat self-harm,^5^ and are recommended by clinical practice guidelines internationally.^23,24^ Limited referral or uptake may reflect several system-level barriers, including under-resourced specialist services, variability in the quality and consistency of psychosocial assessments provided in emergency departments^6^ and fragmented referral pathways,^25^ particularly between acute and community care settings.^26^ Addressing these gaps requires policy attention to workforce capacity, service accessibility and continuity of care across sectors.

More broadly, our findings point to structural and service-level reform. In Australia and the UK, health care sectors largely operate in isolation, often requiring treatment for one condition before accessing care for another. Co-locating primary care and secondary services improves coordination and patient outcomes, while reducing operational costs.^27^ We recommend extending community co-location to include AOD services, which could reduce the number of patients who currently receive only brief or screening-only AOD assessments in the emergency department, decrease substance use and related harms, improve mental health symptoms, lower reliance on crisis services and decrease health system costs.^28^ Introducing cross-sector care coordinators could help bridge fragmentation across sectors by providing patients with a central point of contact, supporting timely engagement with services and reducing the number of patients disengaged from services. Finally, implementing assertive follow-up within 7 days after an emergency department presentation for self-harm could ensure continuity of care, improve linkage to appropriate services and promote sustained engagement with evidence-based services.

### Strengths

Strengths include population-level administrative health data and a novel data-driven approach to capture dynamic changes in health services use during and after an acute emergency department self-harm presentation. Additionally, given known data-quality issues in identifying episodes of self-harm from routine hospital administrative data sources alone,^29^ our cohort was identified from an enriched data source in which self-harm presentations are identified using robust natural language processing and validated through periodic manual auditing.^30^ The proportion of emergency department presentations for self-harm observed in this study is comparable with other Australian estimates,^31^ though slightly lower than some from the UK.^32^ This likely reflects differences in the structure of health care services (including funding models) and case-ascertainment methods between the two countries. The RMH catchment area is broadly representative of Australia’s population in age and sex.^9^ Uniquely, the RMH also provides 24/7 emergency mental health services and extended–hours alcohol, drug and social work support, offering a comprehensive model of acute care which increases the completeness of the treatment service contacts captured in this study. Using sequential pattern mining, we were able to describe typical sequences of care after self-harm and identify clusters of individuals following similar pathways, revealing patterns of repeated or fragmented contact that would not be apparent using other methods.

### Limitations

While younger patients were included, our cohort is primarily composed of older adolescents and adults, reflecting the RMH’s role as a tertiary, adult-only hospital. Only emergency department presentations for self-harm were captured, and some individuals may have experienced prior self-harm episodes before study entry which, in turn, could have influenced cluster assignment. Administrative data does not capture episodes of informal care.^33^ Confounding by indication also cannot be ruled out as those accessing specialist mental health services are typically at higher risk of both all-cause and suicide mortality. This reflects the current health system design, in which demand often exceeds capacity, leading to access being prioritised according to perceived clinical risk. Finally, as our study is observational and relies on administrative data, we cannot draw causal conclusions about the effects of different patterns of service use on patient outcomes; associations may instead reflect underlying differences in patient risk, service availability or other unmeasured factors.

Health service use clusters during and after an emergency department self-harm presentation reflect both system strengths and limitations. While acute responses are evident, they are often short-lived, and many patients either remain disengaged or are following care pathways that do not provide sustained, evidence-based support. Under-resourced sectors, fragmented funding and siloed service funding models all contribute to limit treatment continuity, especially for intersecting mental health and AOD sectors. Our findings highlight that the health care system often fails to meaningfully alter care trajectories following a self-harm crisis. Policy alignment with national recommendations, particularly those promoting integrated care, cross-sector care coordination and assertive post-discharge outreach, could improve linkage between services and sectors, ensuring timely follow-up. These approaches have the potential to shift patients away from repeated patterns of crisis-driven care towards more sustained, evidence-based support.

## Supporting information

10.1192/bjo.2026.12033.sm001 Witt et al. supplementary material Witt et al. supplementary material

## Data Availability

This study used linked administrative data provided by the Australian Institute of Health and Welfare (AIHW) and the Centre for Victorian Data Linkage (CVDL). Researchers may apply for access to source data-sets through the relevant data custodians, subject to approval. All analysis code will be made publicly available within a dedicated repository on the first author’s GitHub.

## Supplementary material

The supplementary material is available online at https://doi.org/10.1192/bjo.2026.12033

## Fig. 1 Long description

The flowchart begins with all-cause encounters totaling 551 692. It then excludes encounters not related to self-harm, amounting to 543 955. The remaining self-harm-related encounters involve 5378 persons and 543 955 presentations. The flowchart further excludes persons unable to be linked to the National Linkage Map, totaling 416, and those whose treatment contacts were outside the study window, totaling 294. The final included group consists of 4668 persons and 1 321 813 treatment contacts.

Navigate back to Fig. 1.

## Fig. 2 Long description

The Sankey diagram illustrates the continuity of treatment contact cluster assignment for individuals who had at least one contact in the year before and after their index emergency department presentation for self-harm. The diagram is divided into three clusters: Mixed services cluster, Pharmacy cluster and Specialist mental health services cluster. Each cluster is represented by different colors: purple for Mixed services cluster, teal for Pharmacy cluster and green for Specialist mental health services cluster. The diagram shows the flow of individuals between these clusters before and after the index self-harm episode. The numbers and percentages of individuals in each cluster are provided: Mixed services cluster with 692 (81.1 percent) before and 195 (46.5 percent) after, Pharmacy cluster with 117 (13.9 percent) before and 214 (51.1 percent) after, and Specialist mental health services cluster with 36 (4.3 percent) before and 44 (32.1 percent) after. The flows between clusters indicate how individuals move between different types of treatment contacts over time.

Navigate back to Fig. 2.

## Table 1 Long description

A table classifying health service contacts by care state and data source. The table has two columns: Care state and Data source(s) and definitions. The Care state column lists seven care states: Community services, Emergency departments, General practitioners, Pharmacy, Specialist alcohol and other drug (AOD) services, Specialist mental health services, and Specialist physical health services. The Data source(s) and definitions column provides specific data sources and definitions for each care state. For Community services, the data sources are Mental Health Community Support Services (MHCSS) and Community Health Minimum Data-set (CHMDS). For Emergency departments, the data source is Victorian Emergency Minimum Data-set (VEMD). For General practitioners, the data source is Medicare Benefits Scheme (MBS). For Pharmacy, the data source is Pharmaceutical Benefits Scheme (PBS). For Specialist alcohol and other drug (AOD) services, the data sources are Alcohol and Drug Information System (ADIS), prior to 2017, and Victorian Alcohol and Drug Collection (VADC), post-2017. For Specialist mental health services, the data sources are Victorian Admitted Episodes Data-set (VAED), Victorian Integrated Non-Admitted Health (VINAH), and Operational Data Store (ODS) for any contact coded as ‘mental health’ and/or with mental health providers (e.g., psychologists, psychiatrists). For Specialist physical health services, the data sources are Victorian Admitted Episodes Data-set (VAED), Victorian Integrated Non-Admitted Health (VINAH), and Operational Data Store (ODS) for any contact coded as ‘physical health’ and/or with physical health providers (e.g., dentists, physiotherapists).

Navigate back to Table 1.

## Table 2 Long description

A table comparing treatment contacts by service and provider during and after an index episode of nonfatal self-harm. The table has 26 rows and 4 columns. Column headers are: Treatment service and provider, Contacts (%), Persons (%), Contacts (%), Persons (%) for During and After. Row labels include various treatment services and providers such as Community services, Emergency departments, General practice, Pharmacy, Specialist mental health services, Specialist alcohol and other drug services, Specialist physical health services, Unknown/not reported, Addictions medicine specialist, Allied health worker, Community/voluntary worker, Counsellor, Dentist, Doctor, not otherwise specified, Emergency medicine specialist, General practitioner, Indigenous health worker, Nurse, Occupational therapist, Pharmacist, Physician, Psychologist/psychiatrist, Social worker, Surgeon, Discipline not stated. Each row lists the contacts and persons percentages for during and after the index episode. Notable trends include high contacts and persons percentages for Pharmacy and Specialist physical health services during the episode, and high contacts percentages for Emergency departments and General practice after the episode.

Navigate back to Table 2.

## Table 3 Long description

The table presents data from a univariate multinomial regression model predicting service use pattern clusters during an episode of non-fatal self-harm presenting to the emergency department of the Royal Melbourne Hospital. The table has 6 columns and 55 rows, including headers. The columns are labeled as follows: 1. Pharmacy + AOD services cluster, 2. Specialist physical health services cluster, 3. Specialist mental health services cluster, 4. Primary care cluster, 5. Pharmacy cluster (reference), and 6. Emergency department cluster. Each column contains data on various factors such as demographic information, physical and mental health conditions, and circumstances of the self-harm episode. The factors are listed in the first column, and the subsequent columns provide the odds ratios (OR) and p-values (p) for each factor’s association with the respective service use clusters. For example, the first row shows data for the factor ‘Age group < youth (reference)’, with odds ratios and p-values for each service use cluster. The table provides a detailed comparison of how different factors influence the likelihood of being assigned to each service use pattern cluster.

Navigate back to Table 3.
