# Supplementary material for: Patterns in health service use during and up to 1 year after an acute episode of hospital-presenting self-harm: data linkage cohort study of over 1.3 million records
Source: BJPsych Open. 2026 Jul 10;12(4):e180. doi: 10.1192/bjo.2026.12033 (PMC13359047; doi:10.1192/bjo.2026.12033)
Supplement: Witt et al. supplementary material [file S205647242612033Xsup001.docx]

**SUPPLEMENTARY DOCUMENT**

**Supplementary Table SD1:** Demographic and clinical characteristics of persons presenting to the emergency department of the Royal Melbourne Hospital following an index episode of non-fatal self-harm between 1 January 2012 and 31 December 2019, by linkage status.

|  |  | | **Not linked**  **(N = 416)** | | | | **Linked**  **(N = 4,962)** | | | | **Value** | | ***p*** | |
| --- | --- | --- | --- | --- | --- | --- | --- | --- | --- | --- | --- | --- | --- | --- |
| **Demographic characteristics** |  | | **Mean** | | **(SD)** | | **Mean** | | **(SD)** | |  | |  | |
|  | Age (years)^a^ | | 31.4 | | (20.6) | | 34.3 | | (14.4) | | -3.83 | | <0.001 | |
|  |  | | **N** | | **(%)** | | **N** | | **(%)** | |  | |  | |
|  | Gender^b^ | |  | |  | |  | |  | | 31.90 | | <0.001 | |
|  |  | *Female* | 283 | | (68.0) | | 2,657 | | (53.2) | |  | |  | |
|  |  | *Male* | 133 | | (32.0) | | 2305 | | (46.5) | |  | |  | |
| **Presentation characteristics** |  | | **Mean** | | **(SD)** | | **Mean** | | **(SD)** | |  | |  | |
|  | Length of stay in ED (minutes)^a^ | | 320.2 | | (226.9) | | 349.5 | | (241.7) | | -2.23 | | 0.026 | |
|  |  | | **N** | | **(%)** | | **N** | | **(%)** | |  | |  | |
|  | Method of self-harm^b^ | |  | |  | |  | |  | | 9.85 | | 0.276 | |
|  |  | *Acute alcohol overdose* | <10 | | - | | <10 | | - | |  | |  | |
|  |  | *Drowning* | <10 | | - | | <10 | | - | |  | |  | |
|  |  | *Firearms and/or explosives* | - | | - | | 12 | | (0.2) | |  | |  | |
|  |  | *Hanging and/or asphyxia* | <10 | | - | | 118 | | (2.4) | |  | |  | |
|  |  | *Intentional drug overdose* | 242 | | (58.6) | | 3078 | | (62.6) | |  | |  | |
|  |  | *Self-poisoning* | <10 | | - | | 98 | | (2.0) | |  | |  | |
|  |  | *Self-cutting* | 122 | | (29.5) | | 1196 | | (24.3) | |  | |  | |
|  |  | *Other methods* | 22 | | (5.3) | | 309 | | (6.3) | |  | |  | |
|  |  | *Not reported* | 15 | | (3.6) | | 138 | | (2.8) | |  | |  | |
|  | Alcohol co-involvement^b^ | |  | |  | |  | |  | | 0.88 | | 0.348 | |
|  |  | *Not reported* | 329 | | (79.1) | | 3818 | | (76.9) | |  | |  | |
|  |  | *Yes* | 87 | | (20.9) | | 1144 | | (23.1) | |  | |  | |
|  | Arrival mode^b^ | |  | |  | |  | |  | | 15.42 | | 0.009 | |
|  |  | *Air ambulance* | - | - | | 47 | | (0.9) | |  | |  | |  |
|  |  | *Road ambulance* | 250 | (60.1) | | 3,254 | | (65.6) | |  | |  | |  |
|  |  | *Private patient transfer service* | - | - | | 21 | | (0.4) | |  | |  | |  |
|  |  | *Police-facilitated transfer* | 22 | (5.3) | | 158 | | (3.2) | |  | |  | |  |
|  |  | *Self-presentation* | 81 | (19.5) | | 839 | | (16.9) | |  | |  | |  |
|  |  | *Not reported* | 63 | (15.1) | | 643 | | (13.0) | |  | |  | |  |
|  | Triage category^b^ | |  |  | |  | |  | | 4.27 | | 0.234 | |  |
|  |  | *Immediate* | 25 | (6.0) | | 327 | | (6.6) | |  | |  | |  |
|  |  | *Emergency* | 50 | (12.0) | | 725 | | (14.6) | |  | |  | |  |
|  |  | *Urgent* | 257 | (61.8) | | 3067 | | (61.8) | |  | |  | |  |
|  |  | *Semi-Urgent / Non-Urgent*^c^ | 84 | (19.0) | | 843 | | (15.9) | |  | |  | |  |
|  | Assessed by emergency mental health staff^b^ | |  |  | |  | |  | | 0.01 | | 0.921 | |  |
|  |  | *Not reported* | 193 | (46.4) | | 2321 | | (46.8) | |  | |  | |  |
|  |  | *Yes* | 223 | (53.6) | | 2641 | | (53.2) | |  | |  | |  |
|  | Disposition destination^b^ | |  |  | |  | |  | | 28.14 | | <0.001 | |  |
|  |  | *Absconded* | 30 | (7.2) | | 316 | | (6.4) | |  | |  | |  |
|  |  | *Home* | 183 | (44.0) | | 1721 | | (34.7) | |  | |  | |  |
|  |  | *Short-stay observation unit* | 142 | (34.1) | | 1731 | | (34.9) | |  | |  | |  |
|  |  | *Admitted – medical/surgical ward* | 21 | (5.0) | | 434 | | (8.7) | |  | |  | |  |
|  |  | *Admitted – mental health ward* | 12 | (2.9) | | 264 | | (5.3) | |  | |  | |  |
|  |  | *Admitted – ward not reported* | 24 | (5.8) | | 465 | | (9.4) | |  | |  | |  |
|  |  | *Custodial institution* | <10 | - | | 15 | | (0.3) | |  | |  | |  |
|  |  | *Residential care institution* | <10 | - | | 15 | | (0.3) | |  | |  | |  |

**Table notes:** ED – emergency department, N – number, SD – standard deviation. ^a^ Independent samples t-test. ^b^ Chi-square test for independence. ^c^ Categories combined to preserve privacy as per AIHW data suppression guidelines.

**Supplementary Figure SD1.** Elbow (Panel A) and shadow (Panel B) plots for determining the optimal number of clusters for care pathways during an episode of self-harm presenting to the Royal Melbourne Hospital, 1 January 2012 to 31 December 2019 (*n* = 2,143).

| **PANEL A:** | **PANEL B:** |
| --- | --- |
| 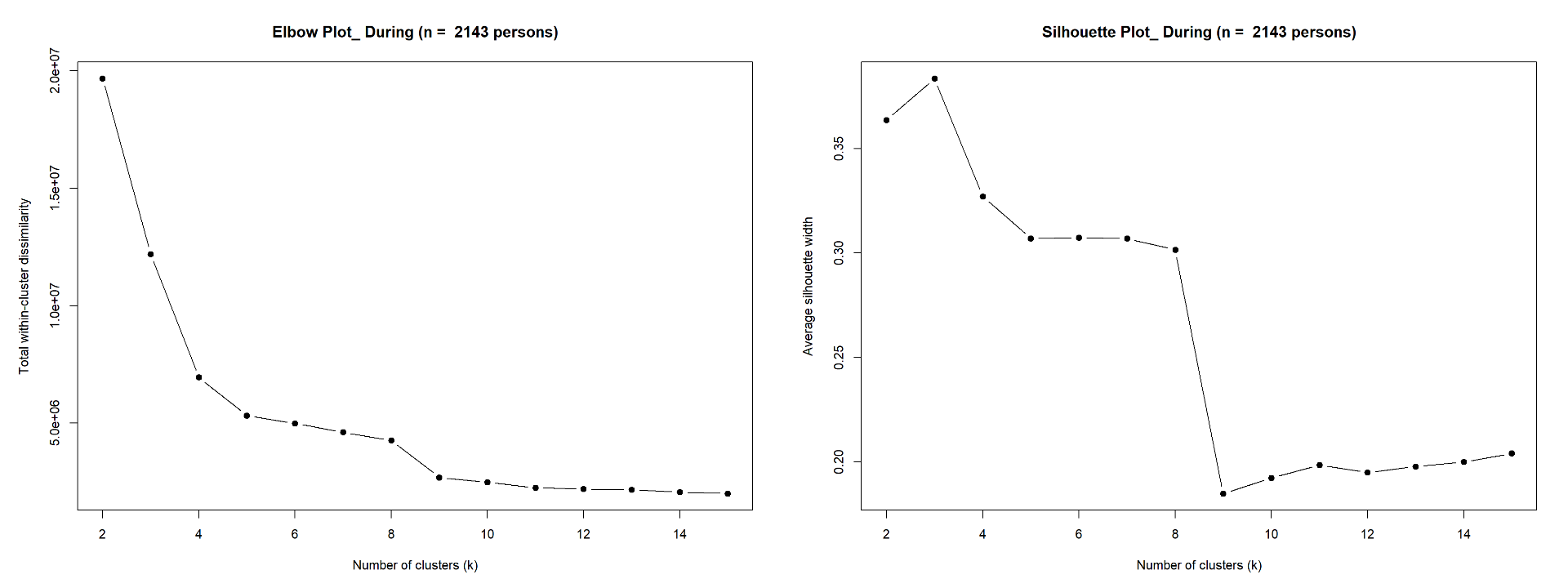 | 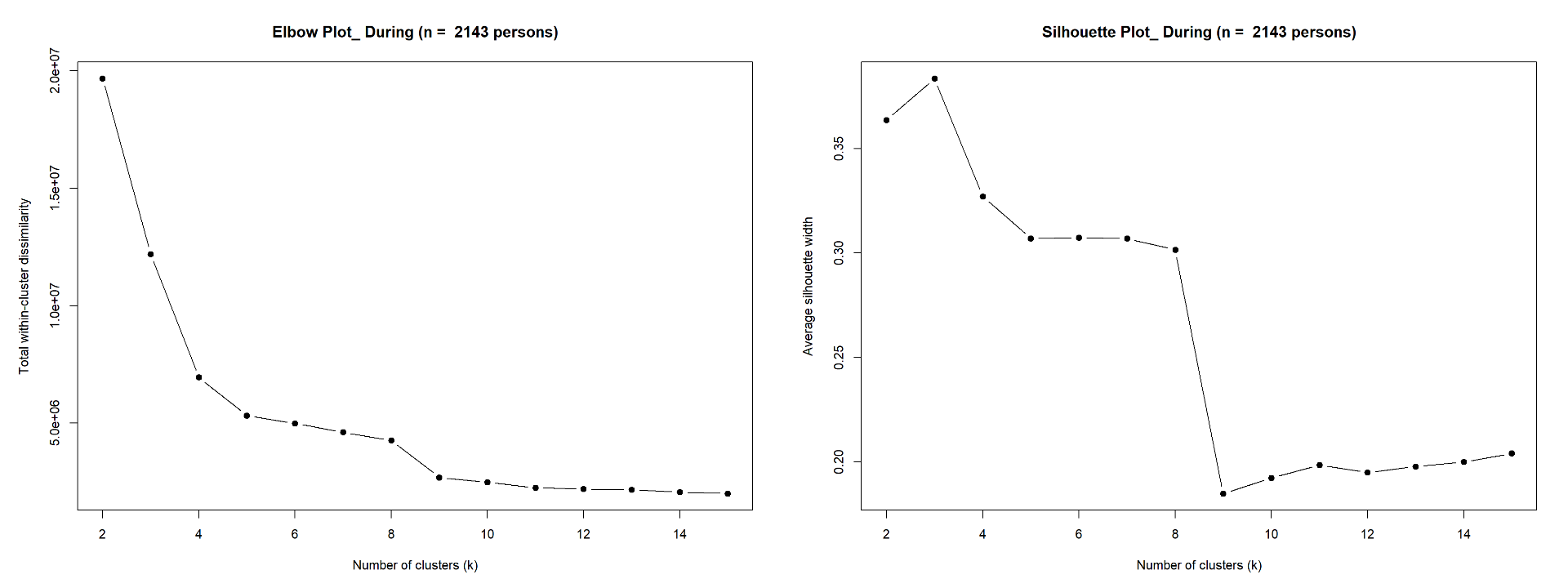 |
| **Elbow plot:** Displays the total within-cluster sum of squares against the number of clusters (*k*). The “elbow” point indicates the optimal number of clusters, balancing cluster compactness with model simplicity. | **Shadow plot:** Shows silhouette widths for each observation across different numbers of clusters (*k*). Higher average silhouette widths indicate better-defined and more cohesive clusters. |

**Supplementary Figure SD2.** Clustered state distribution plot of treatment contacts during acute episodes of ED-presenting self-harm (*n =* 3,653).

|  | Number of Treatment Contacts |
| --- | --- |
|  | 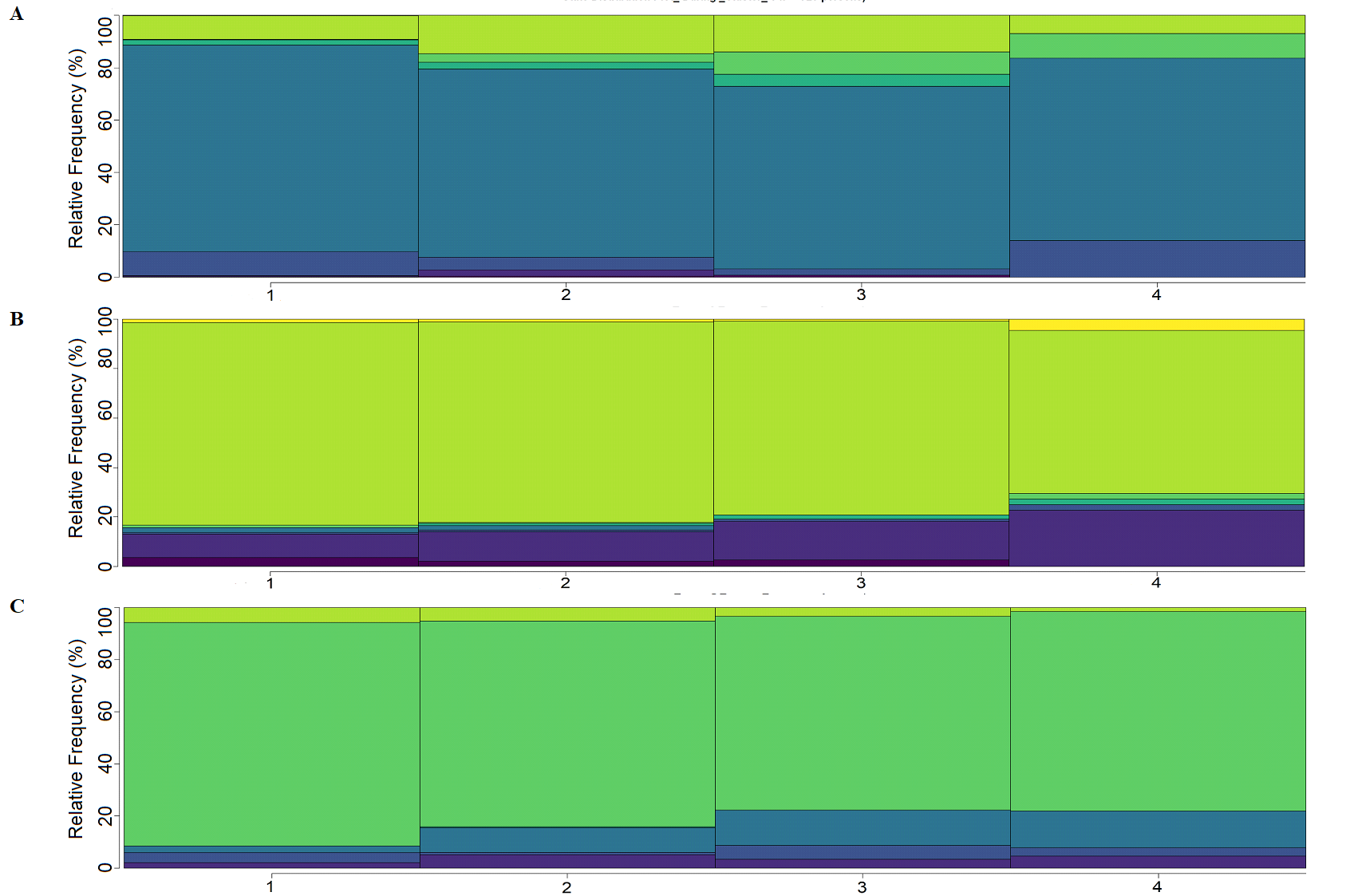 |
| **Pharmacy &** |  |
| **Alcohol & Other** |  |
| **Drug Services** |  |
| **Cluster** |  |
|  |  |
|  |  |
|  |  |
|  |  |
|  |  |
|  |  |
|  |  |
|  |  |
|  |  |
|  |  |
|  |  |
|  |  |
|  |  |
| **Specialist Phys-** |  |
| **ical Health** |  |
| **Services Cluster** |  |
|  |  |
|  |  |
|  |  |
|  |  |
|  |  |
|  |  |
|  |  |
|  |  |
|  |  |
|  |  |
|  |  |
|  |  |
|  |  |
| **Specialist Mental** |  |
| **Health Services** |  |
|  |  |
|  |  |
|  |  |
|  |  |
|  |  |
|  |  |
|  |  |
|  |  |
|  |  |
|  |  |
|  |  |
|  |  |

|  | Number of Treatment Contacts, continued |
| --- | --- |
|  | 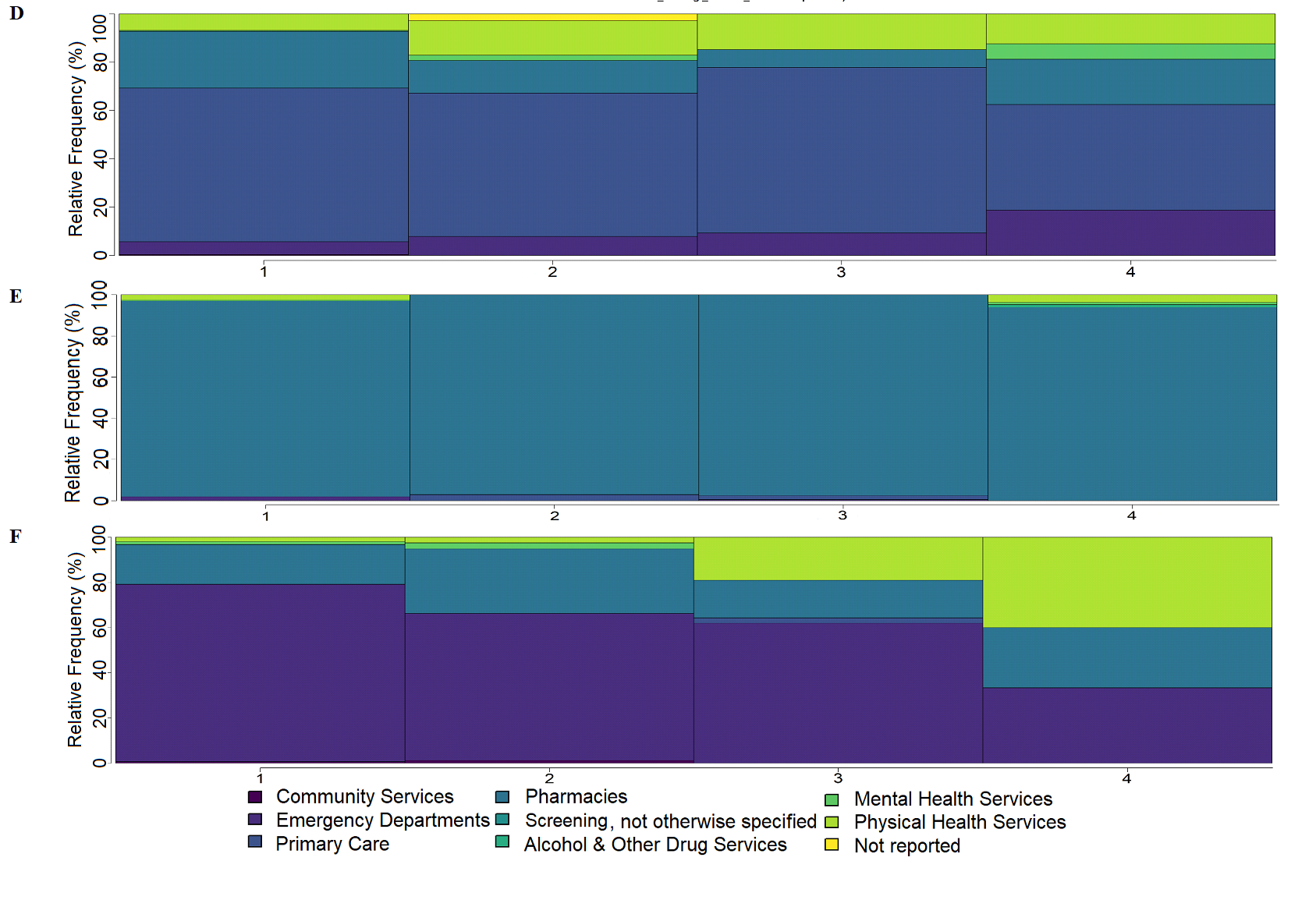 |
| **General Practice** |  |
| **Cluster** |  |
|  |  |
|  |  |
|  |  |
|  |  |
|  |  |
|  |  |
|  |  |
|  |  |
|  |  |
|  |  |
|  |  |
|  |  |
|  |  |
|  |  |
| **Pharmacy** |  |
| **Cluster** |  |
|  |  |
|  |  |
|  |  |
|  |  |
|  |  |
|  |  |
|  |  |
|  |  |
|  |  |
|  |  |
|  |  |
|  |  |
| **Emergency** |  |
| **Department** |  |
| **Cluster** |  |
|  |  |
|  |  |
|  |  |
|  |  |
|  |  |
|  |  |
|  |  |
|  |  |
|  |  |
|  |  |
|  |  |
|  |  |
|  |  |
|  |  |

**Supplementary Figure SD3.** Individual sequence plot of treatment contacts during acute episodes of ED-presenting self-harm (*n =* 100).
Note, sequences generated using synthetic data to preserve privacy as per AIHW data suppression guidelines.

|  | Number of Treatment Contacts |
| --- | --- |
| **Pharmacy &** | 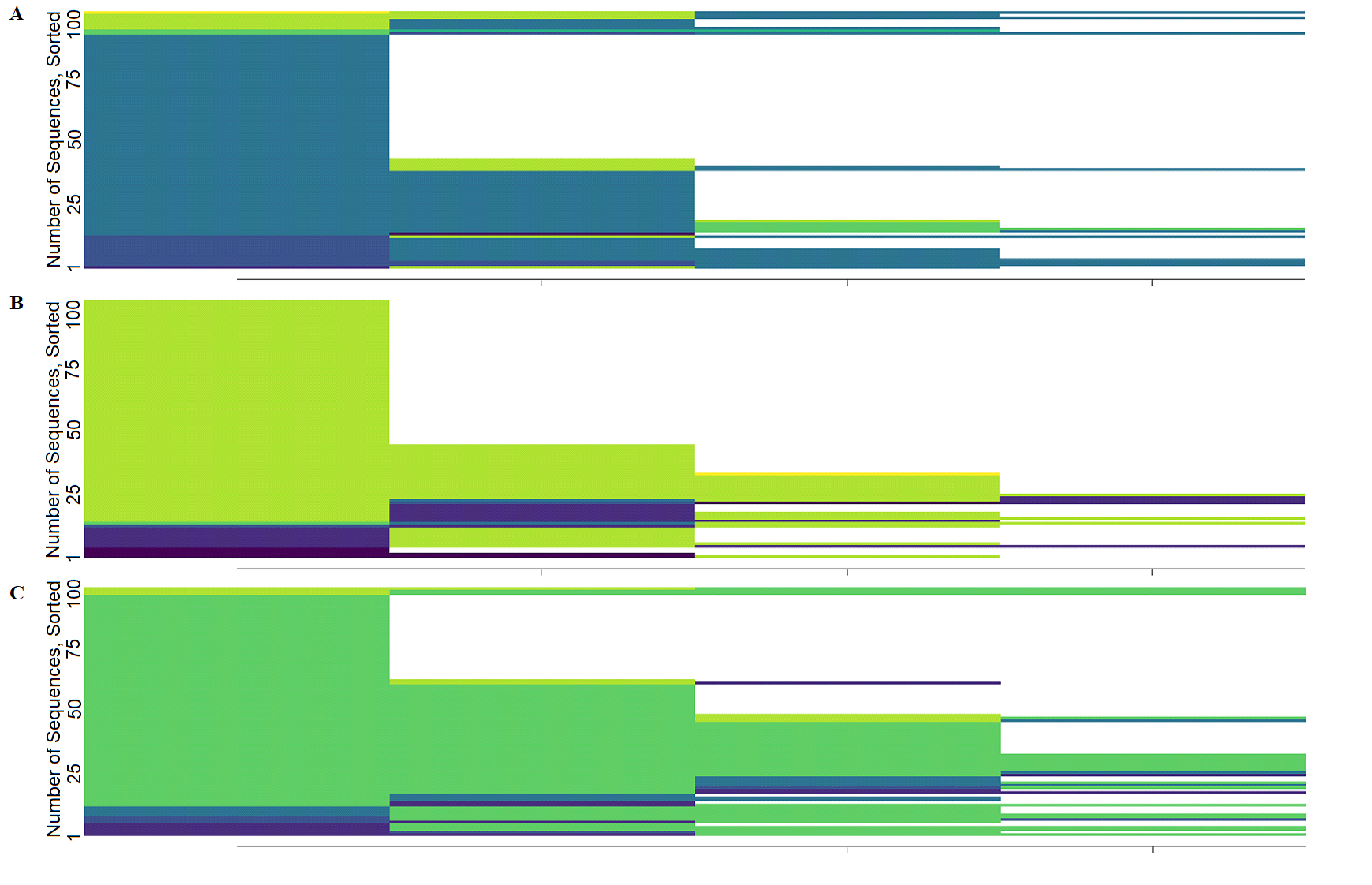 |
| **Alcohol & Other** |  |
| **Drug Services** |  |
| **Cluster** |  |
|  |  |
|  |  |
|  |  |
|  |  |
|  |  |
|  |  |
|  |  |
|  |  |
|  |  |
|  |  |
|  |  |
|  |  |
| **Specialist Phys-** |  |
| **ical Health** |  |
| **Services Cluster** |  |
|  |  |
|  |  |
|  |  |
|  |  |
|  |  |
|  |  |
|  |  |
|  |  |
|  |  |
|  |  |
|  |  |
|  |  |
| **Specialist Mental** |  |
| **Health Services** |  |
| **Cluster** |  |
|  |  |

|  | Number of Treatment Contacts, continued |
| --- | --- |
| **General Practice** | 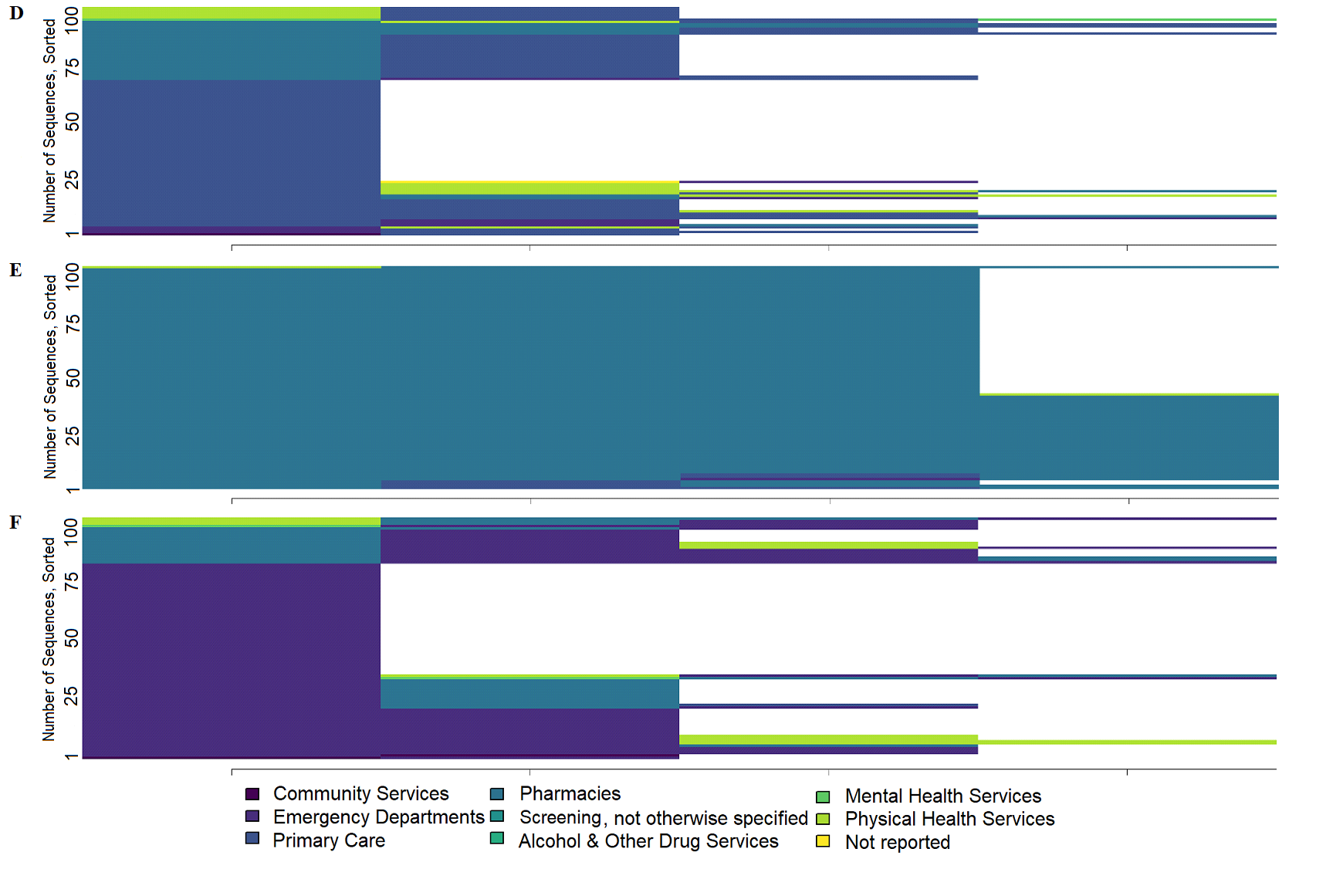 |
| **Cluster** |  |
|  |  |
|  |  |
|  |  |
|  |  |
|  |  |
|  |  |
|  |  |
|  |  |
|  |  |
|  |  |
|  |  |
|  |  |
| **Pharmacy** |  |
| **Cluster** |  |
|  |  |
|  |  |
|  |  |
|  |  |
|  |  |
|  |  |
|  |  |
|  |  |
|  |  |
|  |  |
|  |  |
|  |  |
| **Emergency** |  |
| **Department** |  |
| **Cluster** |  |
|  |  |
|  |  |
|  |  |
|  |  |

**Supplementary Figure SD4.** Elbow (Panel A) and shadow (Panel B) plots for determining the optimal number of clusters for care pathways following an episode of self-harm presenting to the Royal Melbourne Hospital, 1 January 2012 to 31 December 2019 (*n* = 2,304).

| **PANEL A:** | **PANEL B:** |
| --- | --- |
| 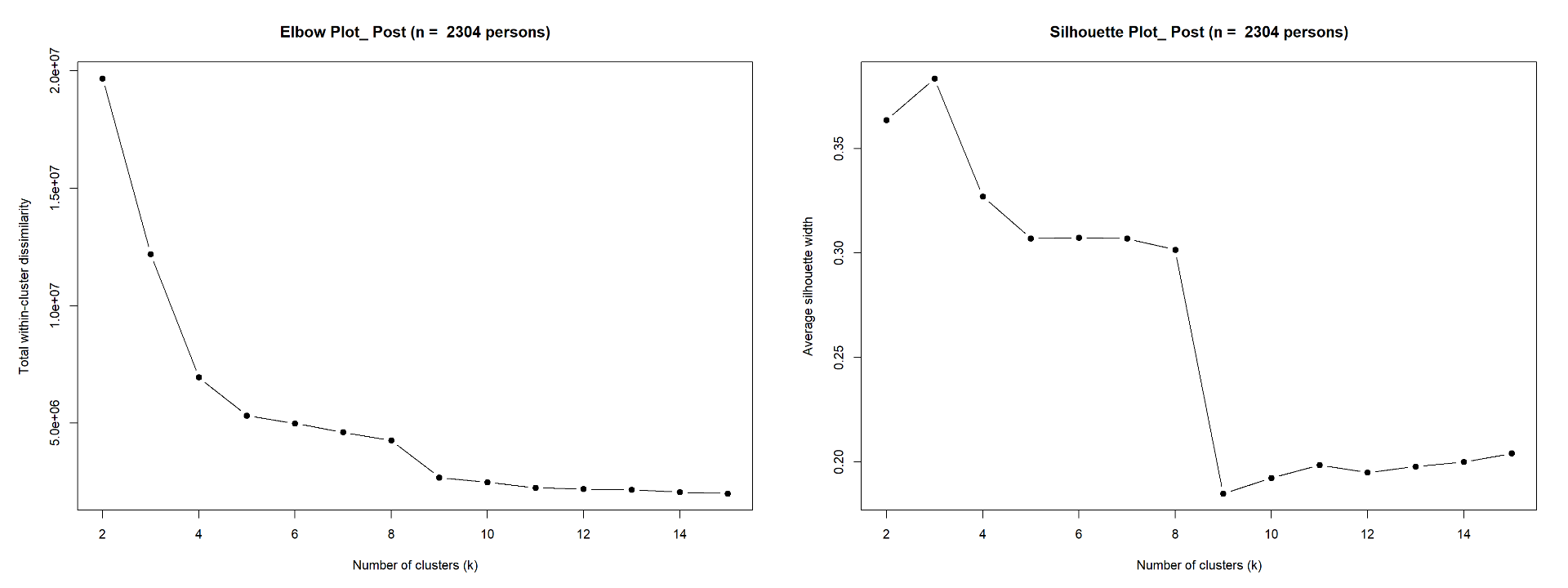 | 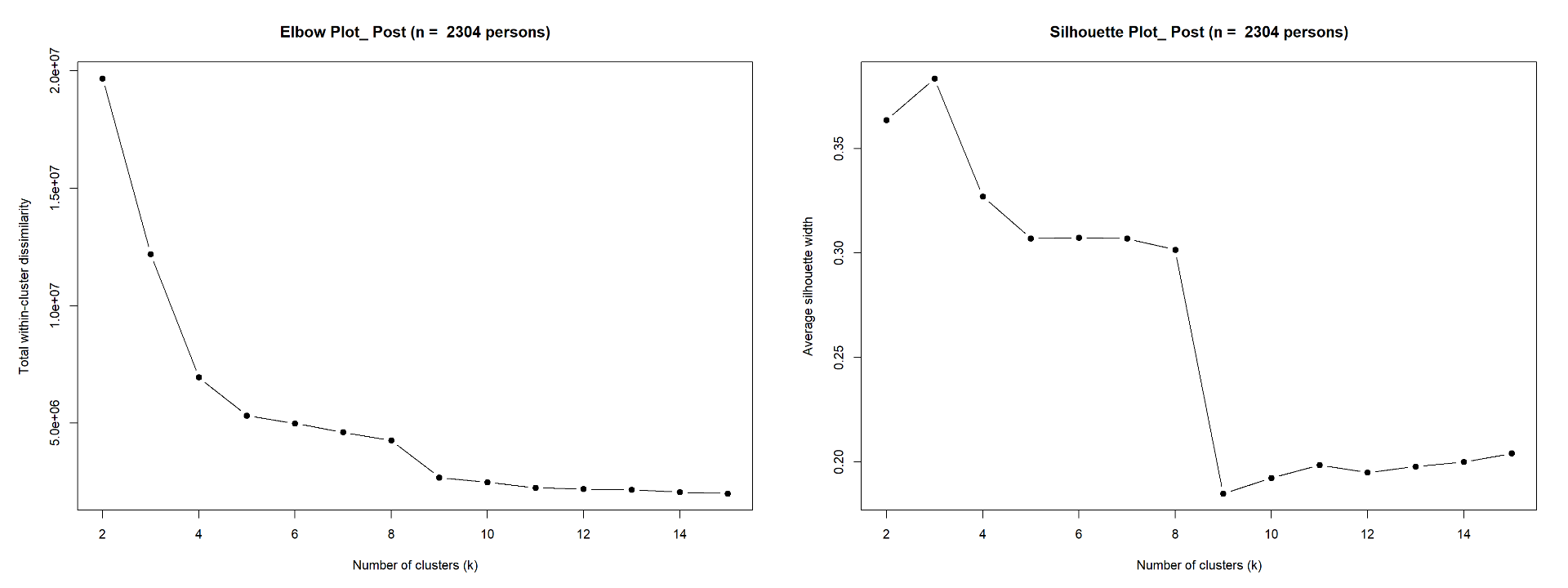 |
| **Elbow plot:** Displays the total within-cluster sum of squares against the number of clusters (*k*). The “elbow” point indicates the optimal number of clusters, balancing cluster compactness with model simplicity. | **Shadow plot:** Shows silhouette widths for each observation across different numbers of clusters (*k*). Higher average silhouette widths indicate better-defined and more cohesive clusters. |

**Supplementary Figure SD5:** Clustered state distribution plot of treatment contacts up to one-year after an index episode of ED-presenting self-harm (*n =* 4,075).

|  | Number of Treatment Contacts |
| --- | --- |
|  | 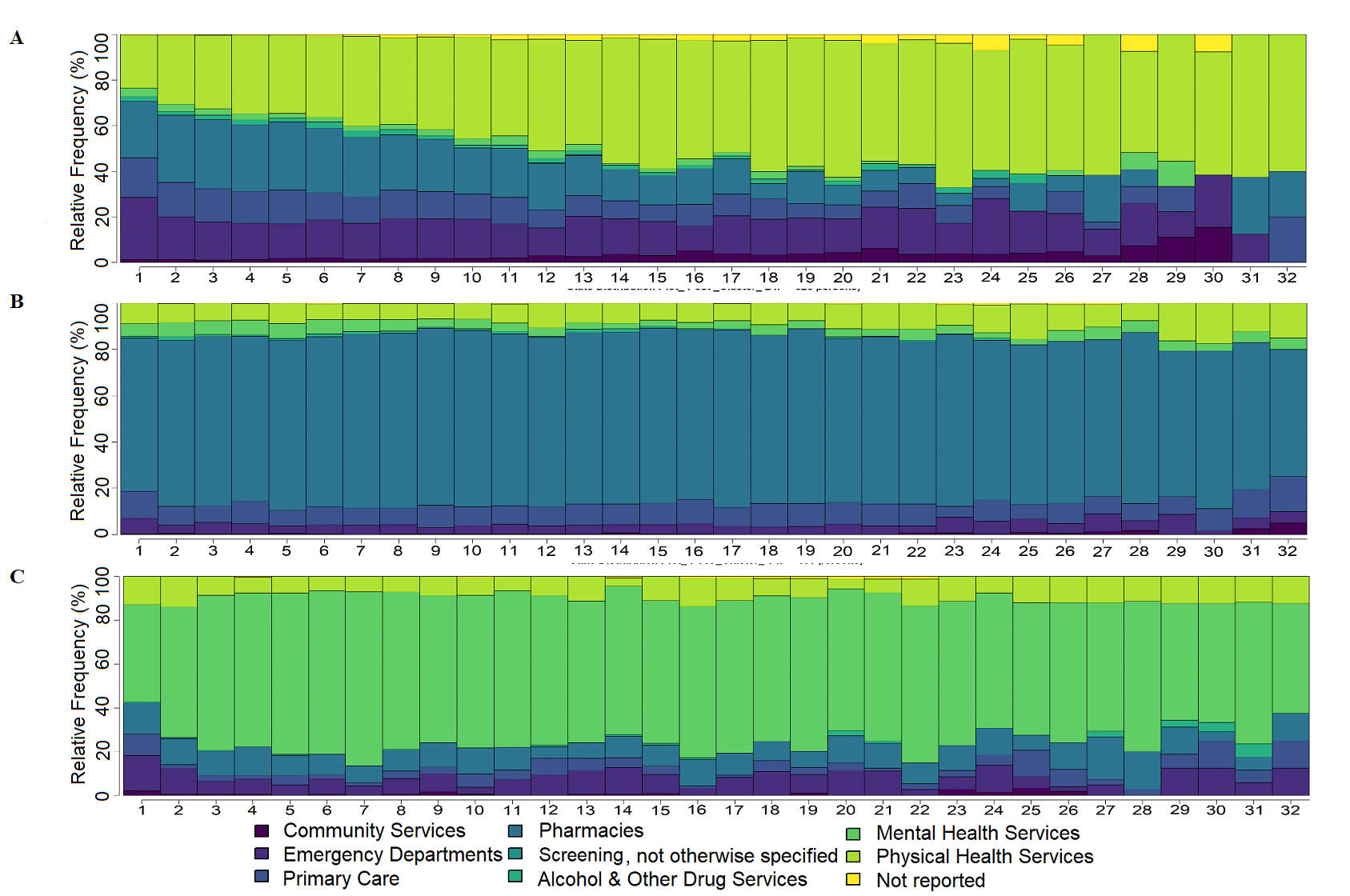 |
| **Mixed** |  |
| **Services Use** |  |
| **Cluster** |  |
|  |  |
|  |  |
|  |  |
|  |  |
|  |  |
|  |  |
|  |  |
|  |  |
|  |  |
|  |  |
|  |  |
|  |  |
|  |  |
| **Pharmacy** |  |
| **Cluster** |  |
|  |  |
|  |  |
|  |  |
|  |  |
|  |  |
|  |  |
|  |  |
|  |  |
|  |  |
|  |  |
|  |  |
|  |  |
|  |  |
| **Specialist Mental** |  |
| **Health Services** |  |
| **Cluster** |  |
|  |  |
|  |  |
|  |  |
|  |  |
|  |  |
|  |  |
|  |  |
|  |  |
|  |  |
|  |  |
|  |  |
|  |  |
|  |  |

**Supplementary Figure SD6:** Individual sequence plot of treatment contacts up to one-year after an index episode of ED-presenting self-harm (*n =* 100). Note, sequences generated using synthetic data to preserve privacy as per AIHW data suppression guidelines.

|  | Number of Treatment Contacts |
| --- | --- |
|  | 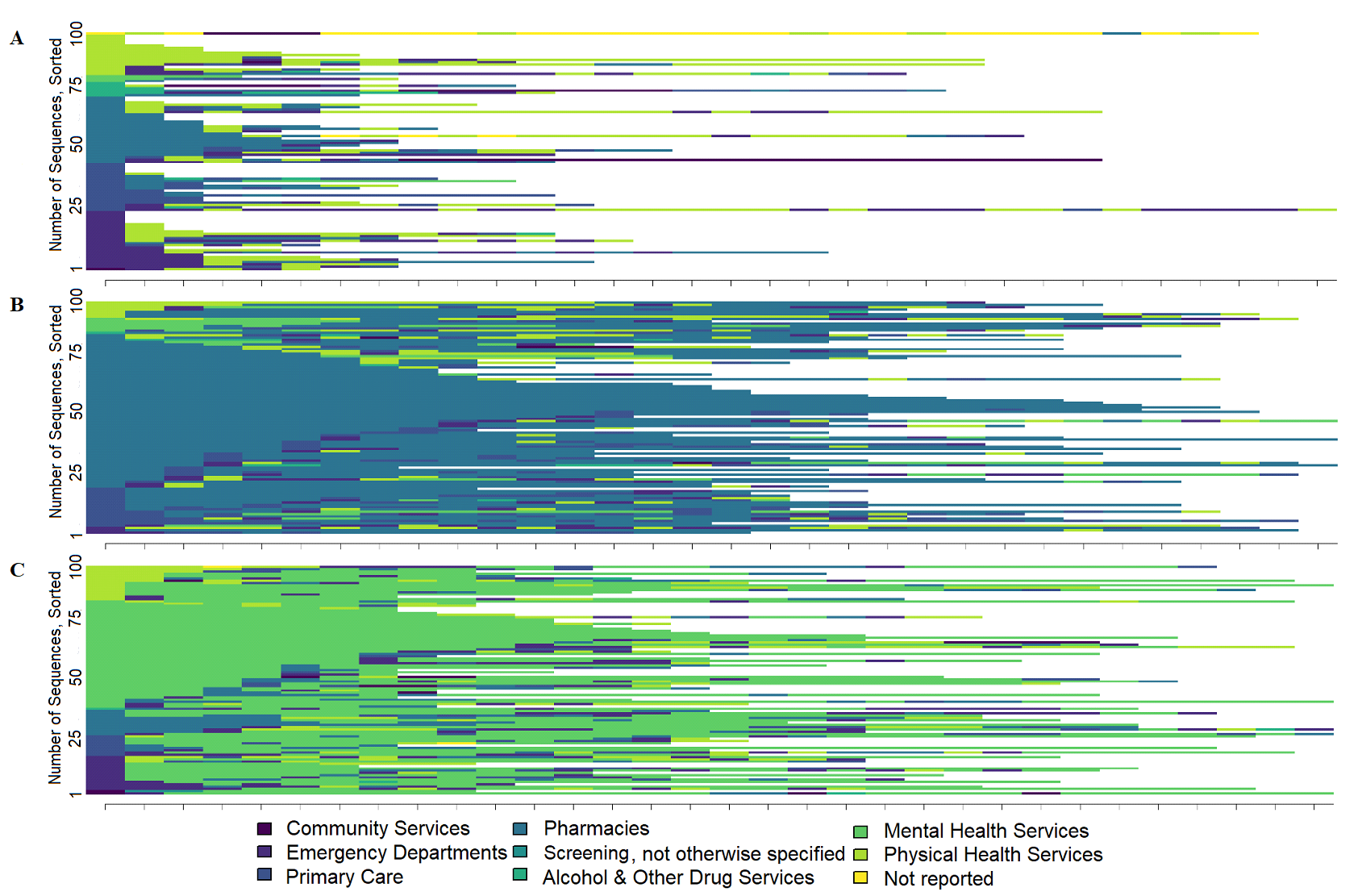 |
| **Mixed** |  |
| **Services Use** |  |
| **Cluster** |  |
|  |  |
|  |  |
|  |  |
|  |  |
|  |  |
|  |  |
|  |  |
|  |  |
|  |  |
|  |  |
|  |  |
|  |  |
|  |  |
| **Pharmacy** |  |
| **Cluster** |  |
|  |  |
|  |  |
|  |  |
|  |  |
|  |  |
|  |  |
|  |  |
|  |  |
|  |  |
|  |  |
|  |  |
|  |  |
|  |  |
| **Specialist Mental** |  |
| **Health Services** |  |
| **Cluster** |  |
|  |  |
|  |  |
|  |  |
|  |  |
|  |  |
|  |  |
|  |  |
|  |  |
|  |  |
|  |  |
|  |  |
|  |  |
|  |  |

***Treatment Service Patterns up to 1 Year Preceding Self-Harm***

Overall, 413 persons (8·8%) had no treatment contacts, while 4,255 (91.2%) had at least one (median 22, IQR 7 to 64). Among those with at least one contact, half (54.0%) were female. The median duration of treatment was six days (IQR 2 to 13), and the median duration between contacts was eight days (IQR 3 to 17). Most had treatment contacts with pharmacy, followed by EDs, and specialist physical health services (Supplementary Table SD2).

**Supplementary Table SD2.** Summary of treatment contacts by service and provider up to one year before an index episode of non–fatal self–harm presenting to the emergency department of the Royal Melbourne Hospital, 1 January 2012 to 31 December 2019.

| Treatment Service and Provider | | BEFORE | |
| --- | --- | --- | --- |
|  |  | **Contacts (%)** | **Persons (%)** |
| Treatment service | |  |  |
|  | *Community services* | 215 (0·9) | 89 (1·5) |
|  | *Emergency departments* | 2,888 (11·9) | 1,372 (23·5) |
|  | *General practice* | 2,077 (8·6) | 969 (16·6) |
|  | *Pharmacy* | 10,094 (41·8) | 1,411 (24·2) |
|  | *Specialist mental health services* | 2,089 (15·8) | 504 (8·6) |
|  | *Specialist alcohol and other drug services* | 252 (1·0) | 126 (2·2) |
|  | *Specialist physical health services* | 4,746 (19·6) | 1,330 (22·8) |
|  | *Unknown/not reported* | 90 (0·4) | 38 (0·7) |
| Treatment provider | |  |  |
|  | *Addictions medicine specialist* | 408 (0·8) | 111 (1·6) |
|  | *Allied health worker* | 4,776 (9·8) | 1,113 (16·1) |
|  | *Community/voluntary worker* | - | - |
|  | *Counsellor* | 378 (0·8) | 76 (1·1) |
|  | *Dentist* | 12 (0·0) | <10 |
|  | *Doctor, not otherwise specified* | 2,554 (5·2) | 511 (7·4) |
|  | *Emergency medicine specialist* | 5,776 (11·9) | 1,372 (19·9) |
|  | *General practitioner* | 4,154 (8·5) | 969 (14·0) |
|  | *Indigenous health worker* | 10 (0·0) | <10 |
|  | *Nurse* | 358 (0·7) | 77 (1·1) |
|  | *Occupational therapist* | 236 (0·5) | 54 (0·8) |
|  | *Pharmacist* | 20,188 (41·5) | 1,411 (20·4) |
|  | *Physician* | 1,060 (2·2) | 354 (5·0) |
|  | *Psychologist/Psychiatrist* | 7,654 (15·7) | 512 (7·4) |
|  | *Social worker* | 98 (0·2) | 27 (0·4) |
|  | *Surgeon* | 668 (1·4) | 222 (3·2) |
|  | *Discipline not stated* | 346 (0·7) | 97 (1·4) |

Clustering identified three distinct patterns (Supplementary Figure SD7): (1) mixed services use (1,425 persons, 60·0%); (2) pharmacy (662 persons, 27·9%), and; (3) specialist mental health services (289 persons, 12·2%) (Supplementary Figures SD8 and SD9). Compared with the pharmacy cluster (reference), a history of cardiovascular disease (CVD), exposure to traumatic experiences, legal problems, and younger age were associated with an increased likelihood of assignment to the mixed services use cluster. Female sex was associated with a reduced likelihood of assignment to either the mixed services or specialist mental health services clusters (Supplementary Table SD3).

**Supplementary Figure SD7.** Elbow (Panel A) and shadow (Panel B) plots for determining the optimal number of clusters for care pathways prior to an episode of self-harm presenting to the Royal Melbourne Hospital, 1 January 2012 to 31 December 2019 (*n* = 2,376).

| **PANEL A:** | **PANEL B:** |
| --- | --- |
| 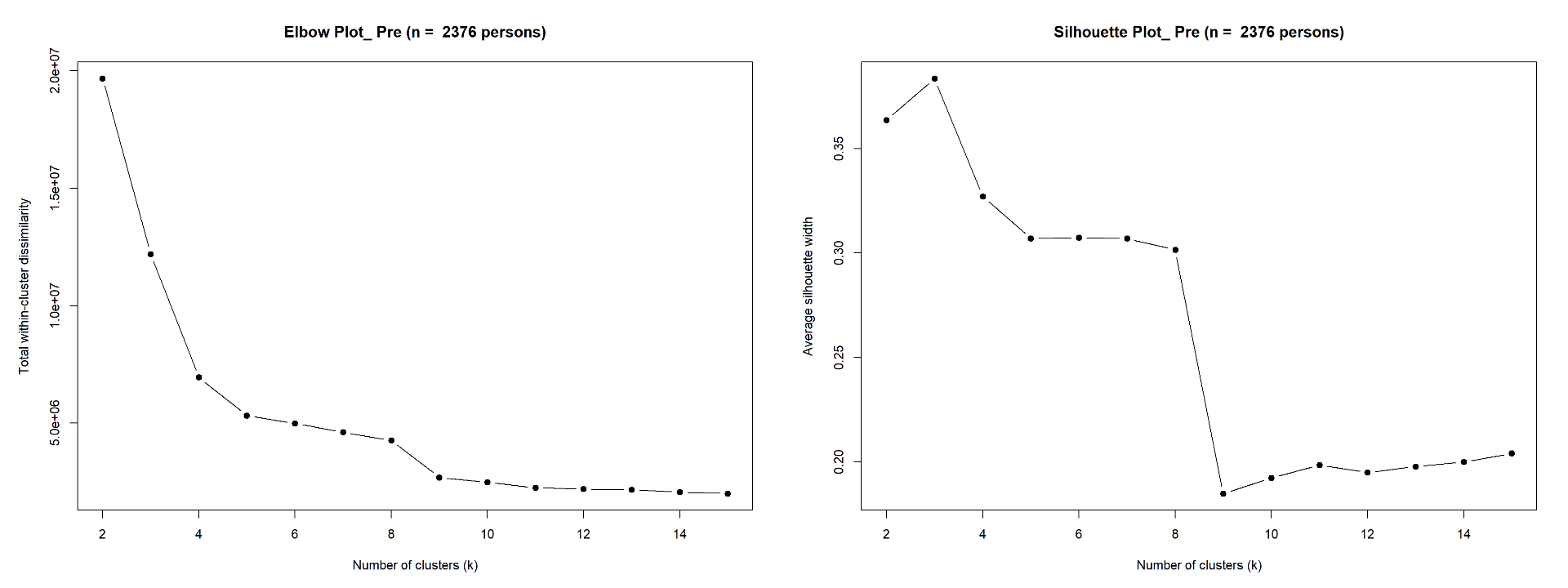 | 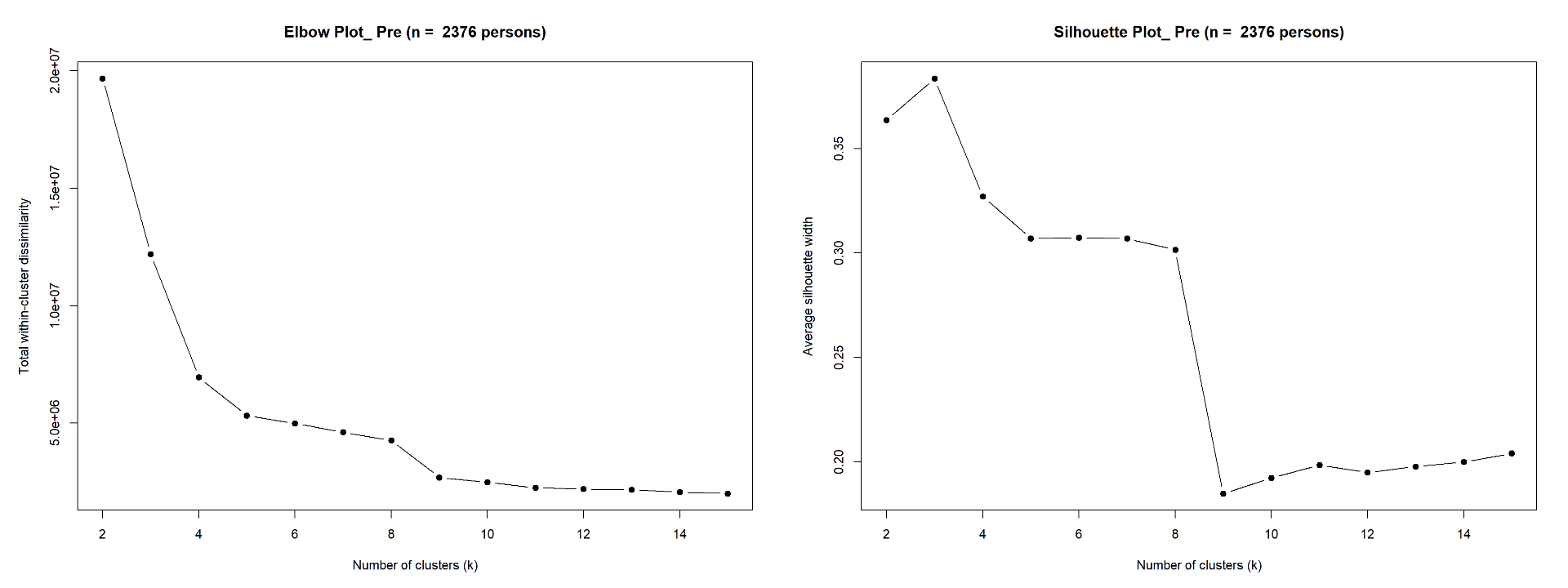 |
| **Elbow plot:** Displays the total within-cluster sum of squares against the number of clusters (*k*). The “elbow” point indicates the optimal number of clusters, balancing cluster compactness with model simplicity. | **Shadow plot:** Shows silhouette widths for each observation across different numbers of clusters (*k*). Higher average silhouette widths indicate better-defined and more cohesive clusters. |

**Supplementary Figure SD8:** Clustered state distribution plot of treatment contacts in the year preceding an index episode of ED-presenting self-harm
(*n =* 4,255).

|  | Number of Treatment Contacts |
| --- | --- |
|  | 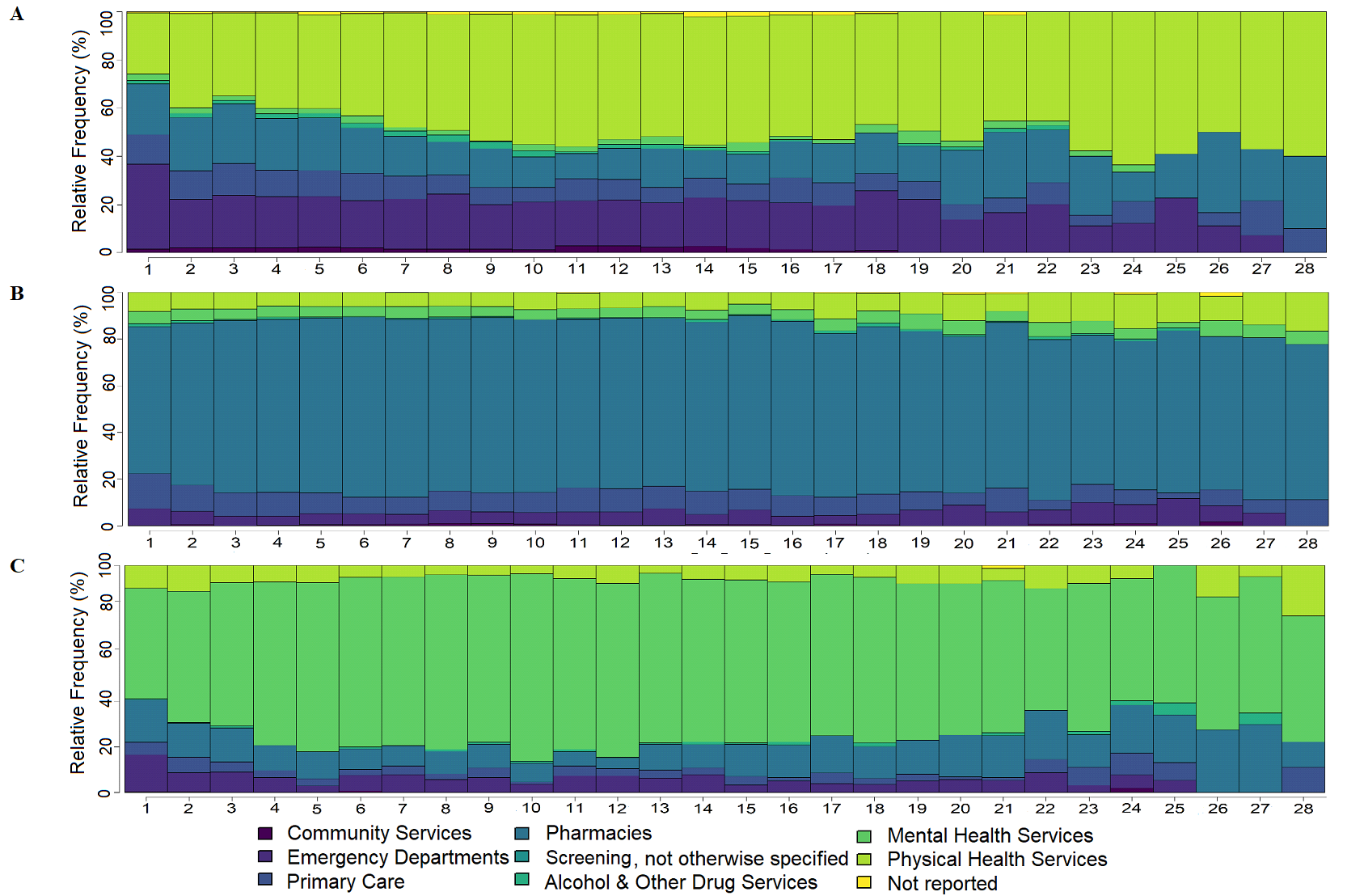 |
| **Mixed Services** |  |
| **Use Cluster** |  |
|  |  |
|  |  |
|  |  |
|  |  |
|  |  |
|  |  |
|  |  |
|  |  |
|  |  |
|  |  |
|  |  |
|  |  |
|  |  |
| **Pharmacy** |  |
| **Cluster** |  |
|  |  |
|  |  |
|  |  |
|  |  |
|  |  |
|  |  |
|  |  |
|  |  |
|  |  |
|  |  |
|  |  |
|  |  |
|  |  |
| **Specialist Mental** |  |
| **Health Services** |  |
| **Cluster** |  |

**Supplementary Figure SD9:** Individual sequence plot of treatment contacts in the year preceding an index episode of ED-presenting self-harm (*n =* 100). Note, sequences generated using synthetic data to preserve privacy as per AIHW data suppression guidelines.

|  | Number of Treatment Contacts |
| --- | --- |
| **Mixed Services** | 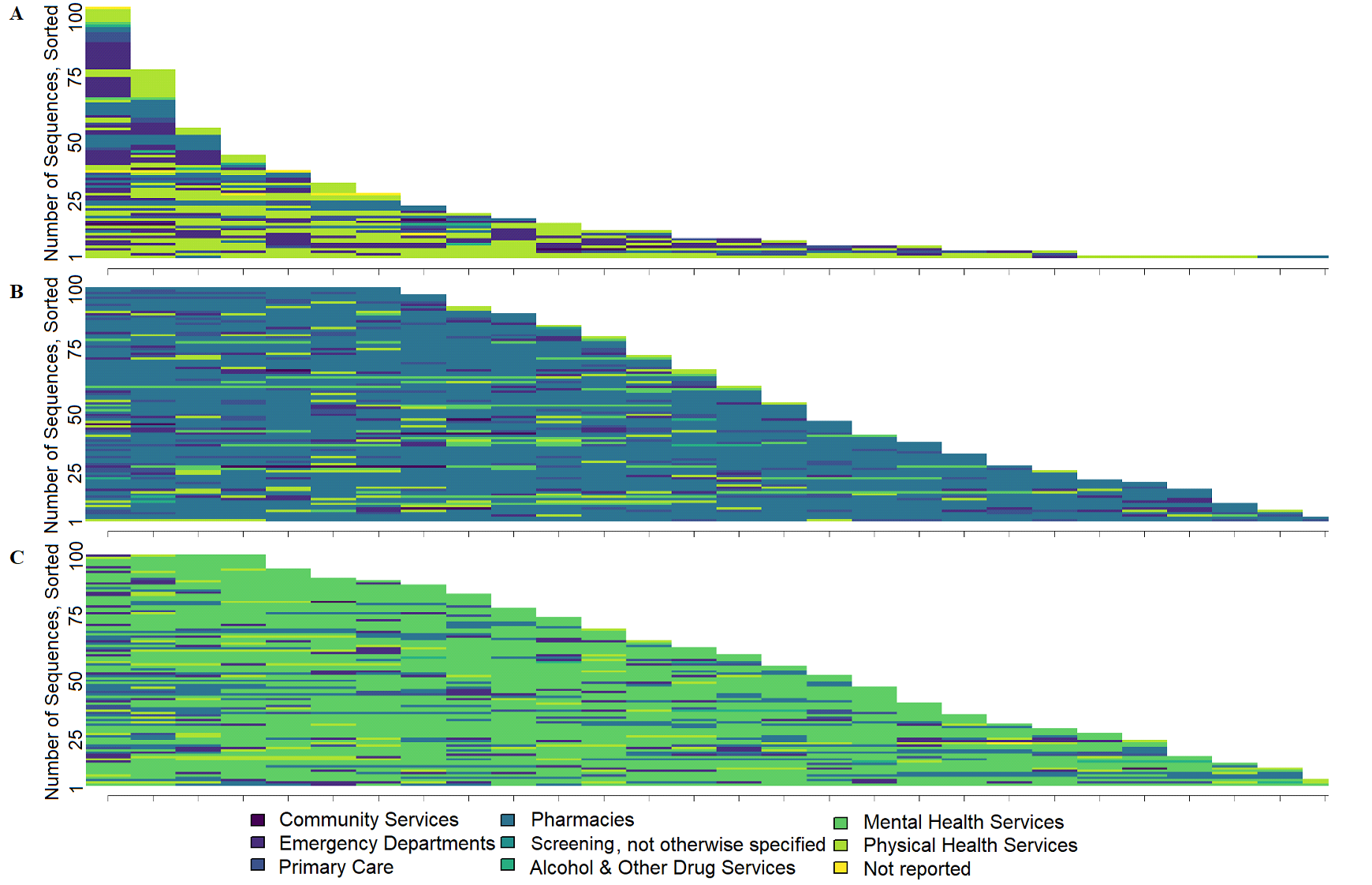 |
| **Use Cluster** |  |
|  |  |
|  |  |
|  |  |
|  |  |
|  |  |
|  |  |
|  |  |
|  |  |
|  |  |
|  |  |
|  |  |
|  |  |
|  |  |
| **Pharmacy** |  |
| **Cluster** |  |
|  |  |
|  |  |
|  |  |
|  |  |
|  |  |
|  |  |
|  |  |
|  |  |
|  |  |
|  |  |
|  |  |
|  |  |
|  |  |
| **Specialist Mental** |  |
| **Health Services** |  |
| **Cluster** |  |
|  |  |

**Supplementary Table SD3.** Univariate multinomial regression models predicting service clusters up to one year before an episode of non–fatal self–harm presenting to the emergency department of the Royal Melbourne Hospital.

| Factor | | 1: Mixed Services Cluster | | | | 2: Pharmacy Cluster  (Reference) | | | | | 3: Specialist MH Services Cluster | | | |
| --- | --- | --- | --- | --- | --- | --- | --- | --- | --- | --- | --- | --- | --- | --- |
|  |  | ***n*** | **OR** | **(95% CI)** | ***p*** | ***n*** | **OR** | **(95% CI)** | | ***p*** | ***n*** | **OR** | **(95% CI)** | ***p*** |
| Demographic | |  |  |  |  |  |  | |  |  |  |  |  |  |
|  | *Age group – youth* | **563** | **1**·**50** | **(1**·**23 – 1**·**82)** | **<0**·**001** | 203 | 1·00 | | (1·00 – 1·00) | NA | 101 | 1·26 | (0·94 – 1·70) | 0·128 |
|  | *ATSI background* | 13 | 1·51 | (0·49 – 4·65) | 0·471 | <10 | 1·00 | | (1·00 – 1·00) | NA | – | – | – | – |
|  | *Sex – female* | **704** | **0**·**61** | **(0**·**50 – 0**·**73)** | **<0**·**001** | 410 | 1·00 | | (1·00 – 1·00) | NA | **127** | **0**·**48** | **(0**·**36 – 0**·**64)** | **<0**·**001** |
|  | *Usual place of residence – regional/rural* | 92 | 0·88 | (0·62 – 1·28) | 0·521 | 48 | 1·00 | | (1·00 – 1·00) | NA | 22 | 1·07 | (0·63 – 1·81) | 0·802 |
| Physical | |  |  |  |  |  |  | |  |  |  |  |  |  |
|  | *No recorded physical illness (reference)* | – | – | – | – | – | – | | – | – | – | – | – | – |
|  | *Cancer* | 15 | 1·11 | (0·66 – 1·86) | 0·693 | 21 | 1·00 | | (1·00 – 1·00) | NA | 10 | 1·09 | (0·51 – 2·35) | 0·818 |
|  | *Chronic pain* | 37 | 1·33 | (0·70 – 2·52) | 0·381 | 13 | 1·00 | | (1·00 – 1·00) | NA | <10 | – | – | – |
|  | *CVD* | **80** | **1**·**91** | **(1**·**15 – 3**·**14)** | **0**·**011** | 20 | 1·00 | | (1·00 – 1·00) | NA | 14 | 1·63 | (0·81 – 3·28) | 0·168 |
|  | *Diabetes* | 68 | 1·23 | (0·77 – 1·94) | 0·387 | 26 | 1·00 | | (1·00 – 1·00) | NA | 10 | 0·88 | (0·42 – 1·84) | 0·729 |
| Psychiatric | |  |  |  |  |  |  | |  |  |  |  |  |  |
|  | *No recorded psychiatric disorder (reference)* | – | – | – | – | –- | – | | – | – | – | – | – | – |
|  | *Anxiety disorder (any)* | 23 | 0·62 | (0·33 – 1·17) | 0·143 | 17 | 1·00 | | (1·00 – 1·00) | NA | <10 | – | – | – |
|  | *Mood disorder (any)* | 118 | 0·75 | (0·55 – 1·02) | 0·071 | 71 | 1·00 | | (1·00 – 1·00) | NA | 31 | 1·00 | (0·64 – 1·56) | 0·999 |
|  | *Eating disorder (any)* | <10 | – | – | – | <10 | 1·00 | | (1·00 – 1·00) | NA | <10 | – | – | – |
|  | *Psychotic disorder (any)* | <10 | – | – | – | <10 | 1·00 | | (1·00 – 1·00) | NA | <10 | – | – | – |
|  | *Personality disorder (any)* | 18 | 1·68 | (0·62 – 4·55) | 0·306 | <10 | 1·00 | | (1·00 – 1·00) | NA | <10 | – | – | – |
|  | *Substance use disorder (any)* | 446 | 1·08 | (0·88 – 1·32) | 0·436 | 196 | 1·00 | | (1·00 – 1·00) | NA | 97 | 1·20 | (0·89 – 1·61) | 0·225 |
| Psychosocial | |  |  |  |  |  |  | |  |  |  |  |  |  |
|  | *No recorded psychosocial factor (reference)* | – | – | – | – | – | – | | – | – | – | – | – | – |
|  | *Exposure to family violence* | 24 | 1·40 | (0·63 – 3·13) | 0·413 | <10 | 1·00 | | (1·00 – 1·00) | NA | <10 | – | – | – |
|  | *Exposure to traumatic experiences* | **127** | **1**·**61** | **(1**·**10 – 2**·**34)** | **0**·**013** | 38 | 1·00 | | (1·00 – 1·00) | NA | 21 | 1·29 | (0·74 – 2·23) | 0·370 |
|  | *Housing problems* | 18 | 1·68 | (0·62 – 4·55) | 0·306 | <10 | 1·00 | | (1·00 – 1·00) | NA | <10 | – | – | – |
|  | *Legal problems* | **128** | **1**·**58** | **(1**·**09 – 2**·**28)** | **0**·**016** | 39 | 1·00 | | (1·00 – 1·00) | NA | 24 | 1·45 | (0·85 – 2·45) | 0·171 |
|  | *Refugee background* | <10 | – | – | – | <10 | 1·00 | | (1·00 – 1·00) | NA | <10 | – | – | – |
|  | *Relationship problems – partner* | – | – | – | – | – | – | | – | – | – | – | – | – |
|  | *Relationship problems – any other person* | <10 | – | – | – | <10 | 1·00 | | (1·00 – 1·00) | NA | <10 | – | – | – |

**Table notes:** ATSI – Aboriginal and/or Torres Strait Islander; CVD – Cardiovascular Disease; MH – Mental Health; NA – not applicable. Dashes indicate categories with no data and/or reference categories. Odds ratios for groups less than 10 suppressed as per AIHW privacy guidelines. Boldface indicates p is significant at conventional <0.05 level.

**Supplementary Figure SD10.** Schoenfeld residuals plots assessing the proportional hazards assumption for cluster assignment at one year (Panel A) and over the entire follow-up period (Panel B) following an episode of self-harm presenting to the Royal Melbourne Hospital, 1 January 2012 to 31 December 2019.

| **PANEL A:** | **PANEL B:** |
| --- | --- |
| 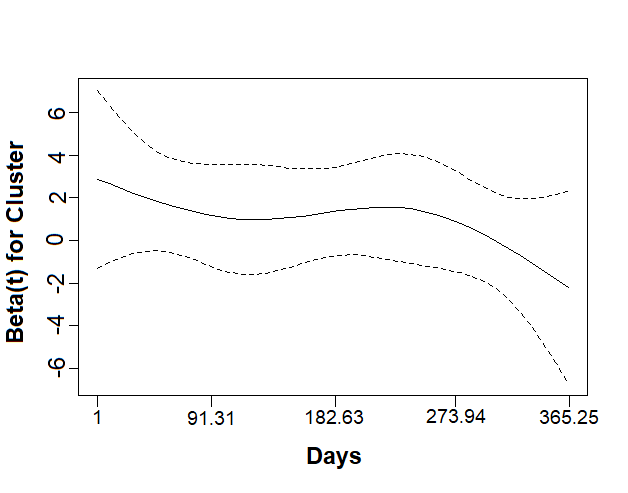 | 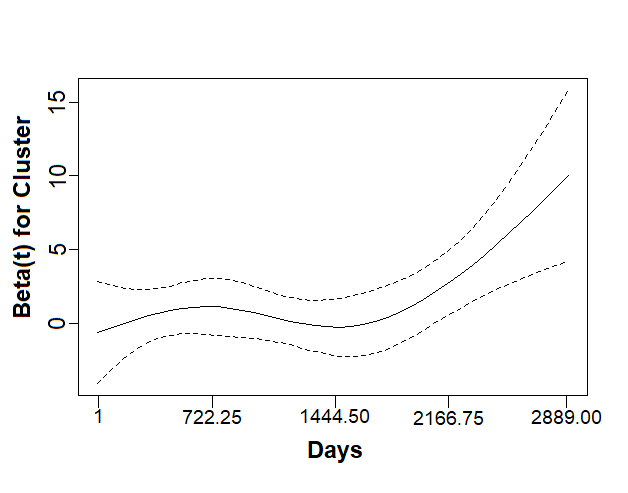 |

**Supplementary Figure SD11.** Cumulative incidence plots for suicide following an episode of self-harm for cluster assignment at one year (Panel A) and over the entire follow-up period (Panel B) following an episode of self-harm presenting to the Royal Melbourne Hospital, 1 January 2012 to 31 December 2019.

| **PANEL A:** | **PANEL B:** |
| --- | --- |
| **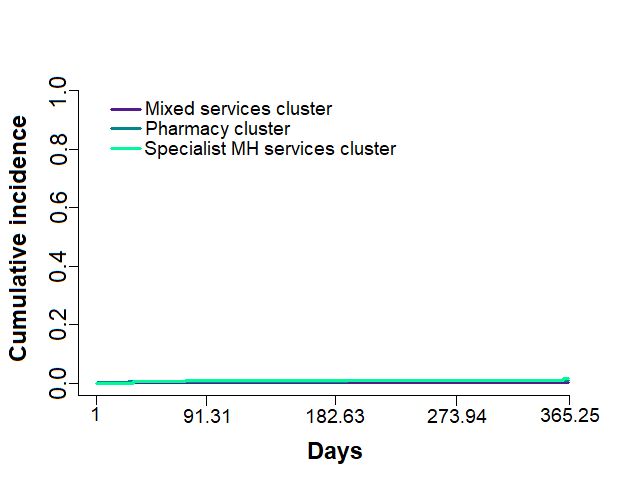** | **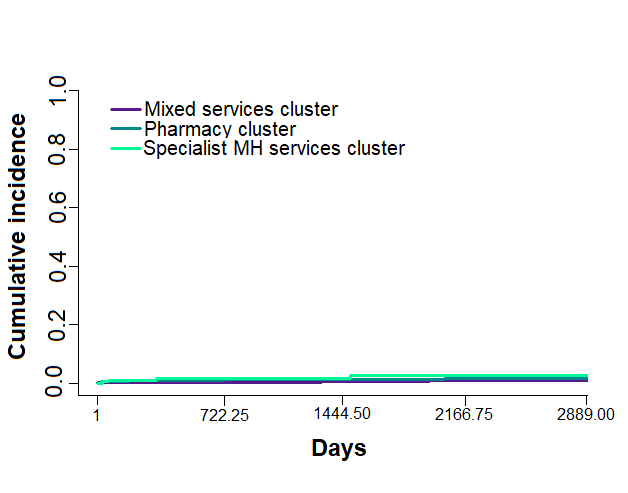** |

**Supplementary Table SD4.** Full description, including study coverage dates, for linked administrative data sources used.

| **Database Name** | **Custodian** | **Linkage Authority** | **Description** | **Study Coverage Dates** | **Summary of  Available Variables** |
| --- | --- | --- | --- | --- | --- |
| **Alcohol and Drug Information System (ADIS)** | Victorian Department of Health and Human Services (DHHS). | Centre for Victorian Data Linkage (CVDL). | Captures data on services by providers funded by the Victorian Government, including AOD treatment services, drink driver assessments and education, and Local Drug Strategy Primary Health Services. | 01/01/2012 – 30/06/2018 (replaced by VADC). | Patient demographics (age, sex, postcode), treatment episodes (start/end dates, service type), primary substance of concern, treatment modality, and service provider details. |
| **Community Health Minimum Dataset (CHMDS)** | Victorian Department of Health (DoH). | CVDL. | Captures data on care provided by community health services, including episodes of allied health, nursing, counselling, and health promotion services. | 01/01/2012 – 31/12/2019. | Patient demographics, type of service provided (e.g., allied health, nursing, counselling), service location, episode start/end dates, and number of contacts. |
| **Medicare Benefits Scheme (MBS)** | Australian Government Department of Health and Aged Care. | AIHW. | Captures data on services subsidised by the Australian Government, including those provided by general practitioners, specialists, and other registered practitioners, including itemised claims for consultations, diagnostics, and procedures. | 01/01/2012 – 31/12/2019. | Patient demographics, service item number, service date, provider type, location, and fee/subsidy amounts. |
| **Mental Health Community Support Services (MHCSS)** | DoH. | CVDL. | Captures data on non-clinical psychosocial and community-based support services provided to people with mental illness, including residential rehabilitation, day programs, and client recovery support packages. | 01/01/2012 – 31/12/2019. | Patient demographics, service type (residential, day program, individual support), referral source, episode dates, and service intensity. |
| **National Deaths Index (NDI)** | Australian Institute of Health and Welfare (AIHW). | AIHW. | Captures data on deaths recorded in Australia. | 01/01/2012 – 31/12/2020. | Patient demographics, date of birth, date of death, state of death registration, and coded cause of death. |
| **Operational Data Store (ODS)** | DoH. | CVDL. | Captures data on specialist mental health services focussed on assessment and management of persons with a mental health condition. | 01/01/2012 – 31/12/2019. | Patient demographics, service encounters, hospital admissions, and clinical status, including diagnosis. |
| **Pharmaceutical Benefits Scheme (PBS)** | Australian Government Department of Health and Aged Care. | AIHW. | Contains data on prescriptions subsidised by the Australian Government and dispensed under the national PBS program. | 01/01/2012 – 31/12/2019. | Patient demographics, medication code, prescription date, supply date, quantity dispensed, prescriber type, and pharmacy location. |
| **Self-Harm Monitoring System for Victoria** | Orygen. | CVDL. | Contains data on episodes of emergency department-presenting self-harm across several hospitals in Victoria. | 01/01/2012 – 31/12/2019. | Patient demographics, date/time of presentation, arrival mode, triage category, method of self-harm, services provided in the emergency department, clinical disposition and location, substances co-involved (alcohol, illicit drugs, and prescription medications). |
| **Victorian Admitted Episodes Dataset (VAED)** | DoH. | CVDL. | Captures data on all hospital admissions to Victorian public hospitals. | 01/01/2012 – 31/12/2019. | Patient demographics, admission/discharge dates, diagnoses (ICD codes), procedures performed, admission type (emergency/elective), hospital code, and discharge outcome. |
| **Victorian Alcohol and Drug Collection (VADC)** | DHHS. | CVDL. | Captures data on treatment episodes for publicly funded alcohol and drug treatment services. | 01/07/2018 – 31/12/2019. | Patient demographics (age, sex, postcode), treatment episodes (start/end dates, service type), primary substance of concern, treatment modality, and service provider details. |
| **Victorian Cause of Death Unit Record File (CODURF)** | Victorian Registrar of Births, Deaths and Marriages. | CVDL. | Contains detailed cause-of-death information for all deaths recorded in the VDI, coded according to the International Classification of Disease, version 10 (ICD-10). | 01/01/2012 – 31/12/2020. | Patient demographics, date of death, place of death, cause-of-death codes (ICD-10), and death registration details. |
| **Victorian Deaths Index (VDI)** | Victorian Registrar of Births, Deaths and Marriages. | CVDL. | Captures data on all deaths recorded in Victoria. | 01/01/2012 – 31/12/2020. | Patient demographics, date of birth, date of death. |
| **Victorian Emergency Minimum Dataset (VEMD)** | DoH. | CVDL. | Captures data on presentations to Victorian public hospital emergency departments. | 01/01/2012 – 31/12/2019. | Patient demographics, date/time of presentation, presenting problem, diagnosis codes, arrival mode, triage category, discharge destination, and hospital code. |
| **Victorian Integrated Non-Admitted Health (VINAH)** | DoH. | CVDL. | Captures data on non-admitted (outpatient and community-based) public health service contacts, including ambulatory care, mental health, and rehabilitation services. | 01/01/2012 – 31/12/2019. | Patient demographics, service type, service date, provider information, episode start/end, and service setting (ambulatory, community, mental health, or rehabilitation). |
